# Biocultural Calendars Across Four Ethnolinguistic Communities in Southwestern South America

**DOI:** 10.1029/2022GH000623

**Published:** 2023-04-20

**Authors:** Ricardo Rozzi, Ricardo Álvarez, Victoria Castro, David Núñez, Jaime Ojeda, Alejandra Tauro, Francisca Massardo

**Affiliations:** ^1^ Cape Horn International Center (CHIC) Omora Ethnobotanical Park Universidad de Magallanes Puerto Williams Chile; ^2^ Sub‐Antarctic Biocultural Conservation Program Department of Philosophy and Religion and Department of Biological Sciences University of North Texas Denton TX USA; ^3^ Cary Institute of Ecosystem Studies Millbrook NY USA; ^4^ Millennium Nucleus Ocean, Heritage & Culture Escuela de Arqueología Universidad Austral de Chile Puerto Montt Chile; ^5^ Departamento de Antropología Facultad de Ciencias Sociales Universidad de Chile Campus Juan Gómez Millas Santiago Chile; ^6^ ONG Poloc Santiago Chile; ^7^ School of Environmental Studies University of Victoria Victoria BC Canada; ^8^ El Colegio de Puebla AC Puebla Mexico

**Keywords:** conservation, ecology, biocultural ethics, worldviews, Chile, climate change

## Abstract

Since the mid‐20th century, the so‐called Great Acceleration (*sensu* Steffen et al., 2007, https://doi.org/10.1579/0044-7447(2007)36[614:TAAHNO]2.0.CO;2) has amplified processes of ecosystem degradation, extinction of biological species, displacement of local peoples, losses of languages, and cultural diversity. These losses are still underperceived by the academic community, and by a global society that is disconnected from biocultural diversity. To reconnect society with biocultural diversity, we integrate temporal and spatial dimensions of seasonal cycles, by combining two conceptual frameworks: ecological calendars and the “3Hs” model of the biocultural ethic (*sensu* Rozzi, 2012, https://doi.org/10.5840/enviroethics20123414). The latter values the vital links between human and other‐than‐human *co‐inhabitants*, their life *habits* (e.g., cultural practices of humans or life cycles of other‐than‐human species), and the structure and processes of their shared *habitats*. This integration enhances an understanding of links between cultural practices and the life cycles of biocultural keystone species. As a synthesis, we use the term *biocultural calendars* to emphasize their co‐constitutive nature that result from interactions between dynamic biophysical and cultural processes embedded in specific ecosystems and cultures. These calendars link astronomical, biological, and cultural seasonal cycles that sustain life and enhance the integration of Indigenous and scientific knowledge to confront challenges of climate change faced from local to global scales. To illustrate this integration, we examine cultural practices and socio‐environmental changes across four contrasting ethnolinguistic communities in southwestern South America, from southern to northern Chile along a marked climatic gradient to show the broad application of the concept of biocultural calendars.

## Key Points

- Biocultural calendars are co‐constitutively generated through interactions between dynamic biophysical and cultural processes
- The biocultural ethic's *3Hs* model values the vital links among human and other‐than‐human *co‐inhabitants*, their *habits*, and shared *habitats*
- Biocultural calendars are based on seasonal cycles of keystone species that are part of communities of co‐inhabitants

## 1 Introduction

**Figure 1:**
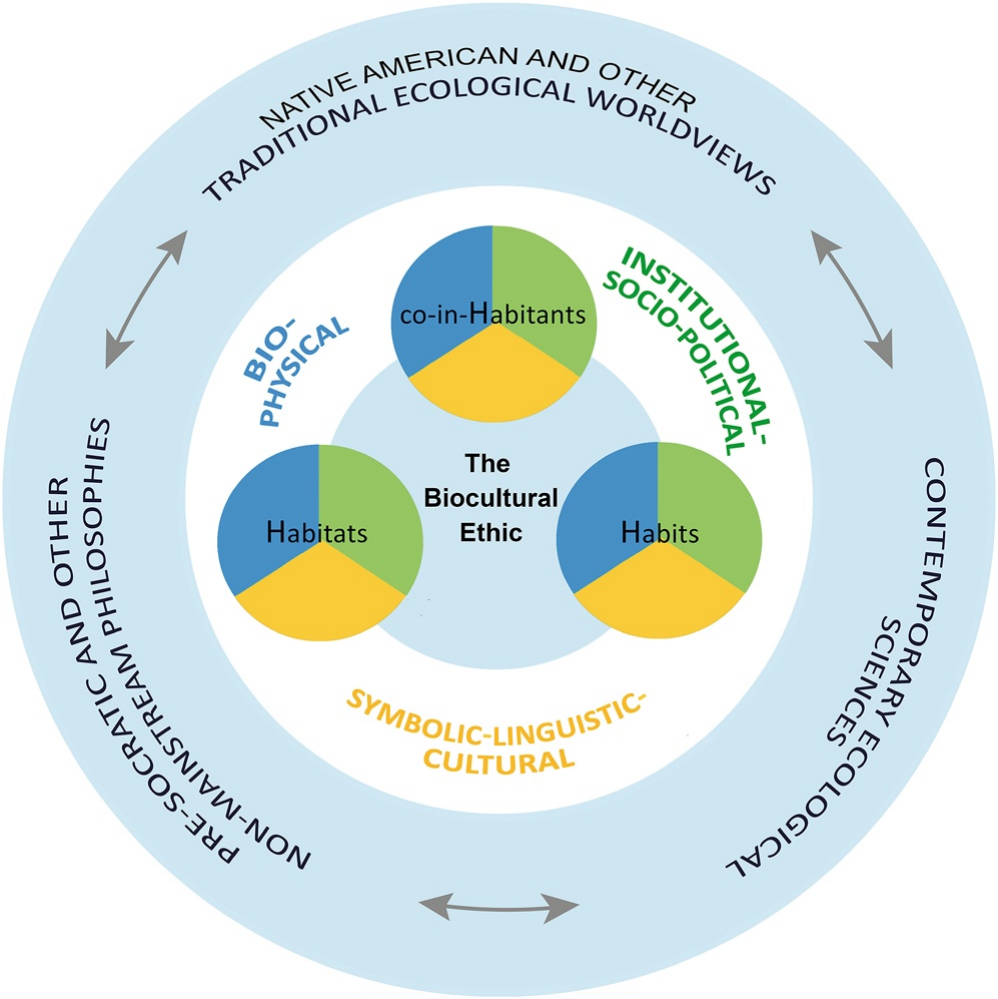
Representation of the “3Hs” (from the Spanish *Habitantes*, *Hábitos*, and *Hábitats*) model of the biocultural ethic that emphasizes the vital links among co‐inhabitants, their life habits and shared habitats. The colors in each circle illustrate that each of the "3Hs" includes biophysical dimensions (blue), symbolic‐linguistic‐cultural dimensions (yellow) and institutional‐socio‐political dimensions (green). The outer circle (in light blue) indicates that the biocultural ethic is informed by a diversity of ancient and contemporary forms of knowledge and worldviews of Indigenous peoples, philosophies, and sciences, along with a plethora of social, political and cultural movements. The bidirectional arrows indicate the multiple active exchanges that occur between worldviews and knowledge in the context of a global society that is dynamic and rapidly changing. Figure modified from Rozzi (2012).

Since the mid‐20th century, the so‐called Great Acceleration (*sensu* Steffen et al., 2007) has amplified processes of ecosystem degradation and the extinction of a growing number of biological species on a global scale (Kolbert, 2011; McNeill & Engelke, 2016). Concomitantly, local peoples and cultures have been displaced from their original habitats, replacing their life habits, and suppressing their relationships with local ecosystems and biological species (Borras et al., 2011, 2012; Bryan, 2012; Rozzi, 2001, 2012). Interrelated losses of biological and cultural diversity have resulted in processes of biocultural homogenization worldwide (Rozzi, 2018). However, it is not the whole human race that is equally responsible for this anthropogenic social‐environmental footprint (Dunn, 2017; Figueroa, 2011; Rozzi, 2007). Consequently, it is necessary to understand sustainable and non‐sustainable social‐environmental impacts generated by specific and distinct human groups, and their practices (Rozzi, 2015b). Identifying the diversity of cultures, their languages, values, and practices in heterogeneous habitats of the planet is a necessary (although not sufficient) step to contribute to reorienting trends of biocultural homogenization toward biocultural conservation, and to move toward sustainability and justice (Rozzi, 2012, 2013, 2018). To undertake this task, and more precisely understand the interrelations between heterogeneous biophysical and cultural dimensions, we use the biocultural ethic's “3Hs” framework that values the vital links among (a) the well‐being and identity of the co‐inhabitants (humans and other‐than‐humans), (The expression “other‐than‐humans” avoids the dichotomy derived from the more usual expression: “non‐humans.” It overcomes this dichotomy for two reasons. First, it alludes to the set of biotic and abiotic beings that form different levels of organization and interactions in the ecosystems they co‐inhabit. Second, the expression “other‐than‐humans” allows us to understand that these beings inhabit not only biophysical nature but also the images, symbols, and values of our cultures. Therefore, they are co‐inhabitants in our biocultural communities, which encompass biophysical and linguistic domains of reality as well as wakeful and oneiric phases of our lives (see Rozzi, 2018), (b) their life habits, and (c) the shared habitats where these vital links take place (Figure 1).

The “3Hs” framework helps to visualize, understand, and value the interdependencies among diverse co‐inhabitants. Understanding and valuing these interdependencies can provide guidance for decision‐making and actions that favor the well‐being of human and other‐than‐human beings. Hence, this framework also has an ethical significance to foster intercultural and inter‐species justice. Conservation of the habitats and access to them is a matter of social‐environmental justice because it is a necessary condition for the continuity of life habits linked to the well‐being and identity of the broad community of co‐inhabitants (Rozzi, 2012).

In this article we use the conceptual frameworks of ecological calendars (Bulbulshoev et al., 2011) and the biocultural ethic (Rozzi, 2012). The first, also known as natural or phenological calendars or seasonal calendars, focuses on the temporal scale of life cycles and other ecological phenomena observed at a given place. Ecological calendars are “knowledge systems to measure and give meaning to time based on close observation of one's habitat” (Kassam et al., 2018, p. 250). Ecological calendars complement the “3Hs” model of the biocultural ethic to better appreciate how Indigenous and other local communities have forms of knowledge, languages, and cultural practices linked to their environment (Dunn, 2017). Across the world, these communities use environmental cues to establish patterns of cultural practices (Ens et al., 2015; Kassam et al., 2011; Singh et al., 2011). Ecological calendars (or “Indigenous seasonal calendars”) are particularly sensitive to climate change both in their biophysical expressions (e.g., earlier flowering phenology of plants or arrival time of migratory birds) and their cultural expressions (e.g., the loss of synchrony between fishing or agricultural practices with life cycles of fishes or plants).

The second conceptual framework, the biocultural ethic considers the interconnectedness between the life cycles of humans and other‐than‐human species and the unique biophysical dimensions of the habitats that they coinhabit. Close observation to biological and cultural diversity, and their interrelationships (in short, biocultural diversity) synchronizes cultural practices with natural processes at specific places. This synchrony is particularly relevant in the context of climate change because it makes explicit the inextricable links between cultures and nature. Changes in cultures involve changes in nature, and vice versa. Communities that are locally attuned to biophysical and cultural habitats have an enhanced capacity to adapt their life habits to variabilities of temperature, rainfall, and other climatic events.

In this article we complement ecological calendars with the biocultural ethic to develop the concept of biocultural calendars. By interrelating phenological events in the biophysical realm with worldviews and practices in the cultural realm, biocultural calendars help us to understand the links between different life habits in contrasting habitats and annual seasons. This attribute of biocultural calendars is crucial in the context of climate change. To develop this concept, we organize our article in two parts. First, we describe the biocultural calendars of Indigenous and other local communities from four climatically contrasting regions of Chile. Second, we concisely discuss the broader implications of this biocultural systemic, contextual, and co‐evolutionary understanding. We propose that biocultural calendars could help set social‐environmental priorities, policies, and actions to conserve critical habitats and biocultural diversity not only in Chile but also in other heterogeneous regions of the planet. We show that our biocultural outlook introduces an understanding that transcends the purely descriptive plane, since it implies an ethical responsibility.

### 1.1 Biocultural Calendars in Chile

In Latin America, the history of Indigenous peoples and local communities is one in which their life habits have fostered co‐inhabitation with high levels of biodiversity (Toledo, 2001; Toledo & Barrera‐Bassols, 2008). Globally, diverse communities cultivate forms of earth stewardship that involve multiple forms of co‐inhabitation with other entities through close material (e.g., food) and symbolic (e.g., mythical actors of social life) ties (Dawson et al., 2021; Rozzi, 2015a). These forms of co‐inhabitation include cultural practices that are closely coupled with the cycles of nature (solar, lunar, or by the seasonal behavior of species related to their subsistence) (Bulbulshoev et al., 2011; Cochran et al., 2016). These biocultural calendars enable communities to meet their needs and collectively cope with unexpected events, such as periods of drought or torrential rains (Kassam et al., 2018). For example, it is possible to anticipate unforeseen changes by observing the behavior of animal species (birds, fish, among others) or the patterns of the landscape (e.g., changes in sea temperature) (Prober et al., 2011; Ryan, 2013). Interestingly, natural scientists have also highlighted the value of not only using the Gregorian year calendar but also ecological calendars (Balvay, 1991).

These environmental phenomena are imprinted in the biocultural oral memory of communities. These memories integrate both biophysical and cultural dimensions of reality (Rozzi, 2015a). Ecological calendars have both biophysical and cultural dimensions that synchronize the rhythms of the natural world or cosmos with the rhythms of human daily activities and rituals. Thus, we use the term *biocultural calendars,* an expression recently used by Mariana Landwehr (2019) on her master's thesis based on a study with the Zinancontec Maya community in Chiapas, Mexico. Maize (*Zea mays* L.) in Zinacantán is part of the cultural identity and is present in the social, political, religious, ceremonial, ritual and gastronomic organization during a well‐defined biocultural calendar (Landwehr, 2019). Complementarily, the term *ecocultural calendars* has been used in participatory biocultural conservation projects (Ali, 2015; Belay, 2012; Flick, 2021). As compared to ecological calendars, both biocultural calendars and ecocultural calendars have the advantage of emphasizing the co‐constitutive processes that generate these calendars in continuous interactions among dynamic biophysical processes, cultural imagination, and traditions of local communities.

We prefer to use biocultural calendars instead of ecocultural calendars because it includes broader biological structures and processes (e.g., the human body, the embodied mind, and common cellular structures shared by eukaryotes) (Rozzi, 2013). Additionally, the term biocultural calendars is consistent with our work, and that of others, on biocultural stewardship (Rozzi, 2015a). More importantly, the term biocultural calendars brings together concepts from ecological calendars as well as from the biocultural ethic and biocultural conservation. This synthesis is relevant for integrating Indigenous and scientific knowledge in order to respond to the complex challenges confronted by local communities and global society in the face of climate change (see Dunn, 2017).

**Figure 2:**
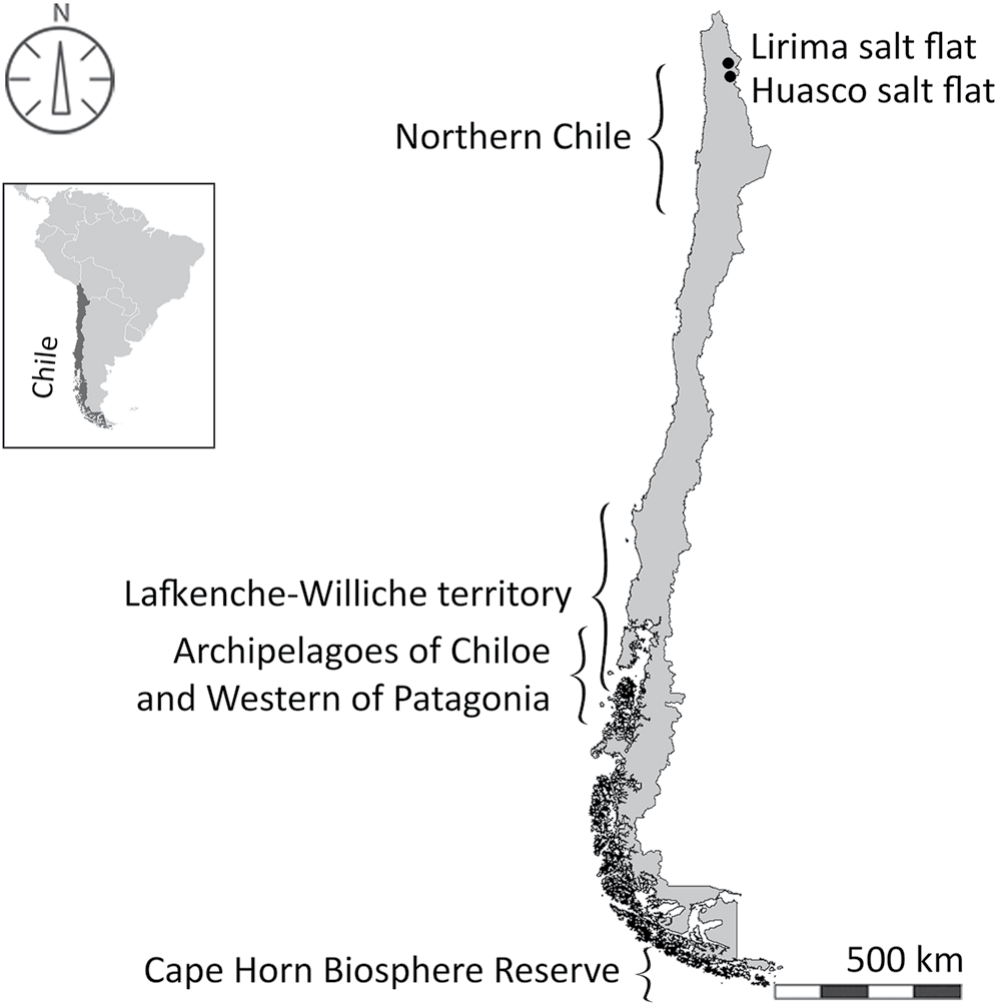
Map of Chile indicating the territories of the biocultural calendars examined in this article. From south to north: Cape Horn Biosphere Reserve (54°–56°S); southwestern Patagonian channels and fjords, including the archipelago of Chiloe (46°–42°S); Lafkenche‐Williche coastal territories in south‐central Chile (43°–35°S); and Salar de Huasco and Lirima high‐Andean wetlands in the extreme north of Chile (21°–17°S).

In this section we use the concept of biocultural calendars to examine cultural practices and socio‐environmental changes in southwestern South America, specifically in Chile (Figure 2). We organized the case studies from the south to the north of the country, spanning 40 degrees of latitude. We start with Cape Horn in the far south where sub‐Antarctic habitats have been co‐inhabited by Yahgan Indigenous peoples for about 10,000 years (Borrero, 2001), and more recently by local fishing communities from Cape Horn to Chiloe (Álvarez et al., 2016; Pyne & Isabella, 2021). Then, the coastal and forests habitats of central‐southern Chile have been co‐inhabited by Lafkenche and Williche communities of the Mapuche Indigenous culture (Álvarez, 2016; Rozzi, Massardo, Anderson, et al., 2010). The extreme north of Chile, at the borders with Bolivia and Peru, is characterized by high Andean habitats of salt flats and wetlands that have been co‐inhabited by Aymara Indigenous communities along with domesticated camelids and a rich biodiversity (Castro & Romo, 2006).

**Figure 3:**
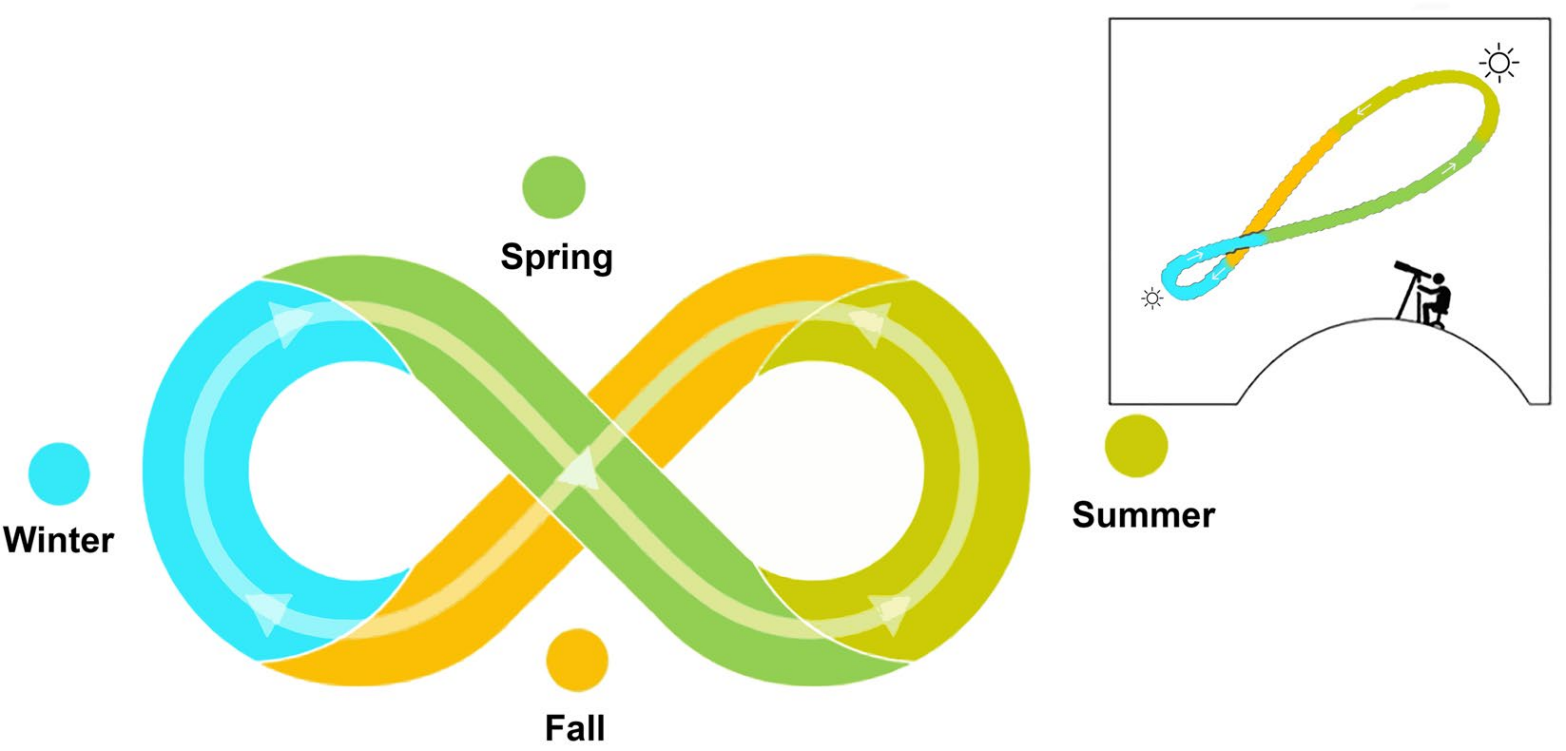
Diagram resembling a solar analemma (insert upper right) to illustrate the dynamic character of biocultural calendars that couple astronomical, biological, and cultural cycles. Each color identifies a season of the year. The insert shows how analemmas are created by observing and capturing the position of the sun in the sky as seen from a fixed location on Earth at the same mean solar time, as that position varies during the course of a year.

To illustrate biocultural calendars, we use a diagram that resembles *analemmas* (or *lemniscates*), showing the position of the sun in the sky as seen from a fixed location on Earth at a fixed time (Figure 3). Our diagrams illustrate the dynamic character of astronomical annual cycles coupled with the cycles of renewal of biotic life and with cycles of cultural practices. Such cycles have been observed in temperate and cold zones with marked changes in temperature and day length between seasons of the year. However, these cycles are also observed in equatorial zones with less evident seasonality but with clear patterns related to the rainy season and other climatic phenomena that inform decision‐making to plant, cultivate, hunt and other cultural practices. Indeed, cyclicality has been observed by diverse Indigenous people who have established biocultural calendars synchronized with the movement of the sun (and the moon) that influence the behavior of the tides and the seasonal cycles of their local climate, plants and animals (Hamacher et al., 2020; Hoffecker, 2005; McHugh et al., 2021).

## 2 Biocultural Calendars at the Southern End of the Americas

### 2.1 Habitats

#### 2.1.1 Biophysical Habitat

The archipelagoes of the southern end of South America have been inhabited for at least 10,000 years (Vásquez & Borrero, 2021). South of Tierra del Fuego, the archipelagoes of the Cape Horn region in Chile host the world's southernmost forests embedded in a mosaic of extensive peatlands (Buma et al., 2020; Rozzi et al., 2012). The islands are surrounded by belts of kelp forests that host a rich marine biodiversity, including edible fish and shellfish (Mansilla et al., 2012). The climate is oceanic with even distribution of monthly rainfall and low temperature amplitude (less than 10°C) (Rozzi et al., 2012). Annual mean temperature is 8.9°C, and rainfall varies between 5,000 mm (at western sites exposed to the Pacific Ocean) and 500 mm (at eastern sites) (Rozzi et al., 2020). The seasonality is characterized by marked changes in the daylength, which influence the phenology of the sub‐Antarctic forests that include broadleaf forests, with evergreen and deciduous species of *Nothofagus* (Veblen et al., 1996). Seasonality, also involve migration of birds and marine mammals. In the seascapes and landscapes of the Yahgan people, spring marks the beginning of the reproductive season and arrival of migratory birds bringing wellness for the communities (McEwan et al., 2014). This area constitutes one of the Earth's most pristine ecoregions (Mittermeier et al., 2003). Today, this remote region of fjords, mountains, glaciers, sea, and intact natural landscapes represents a cultural and natural heritage for all humanity protected as UNESCO Cape Horn Biosphere Reserve) (Rozzi et al., 2006).

#### 2.1.2 Symbolic Linguistic Habitat

The Cape Horn archipelagoes are the ancestral territory of the Yahgans, the world's southernmost Indigenous group (McEwan et al., 2014). Nomadic hunters, fishers, and gatherers, they canoed the channels of these archipelagoes south of Tierra del Fuego, leaving behind a remarkable legacy of material and immaterial culture (Gusinde, 1961). Hundreds of shell middens found along the coasts testify to their material culture (Rivas et al., 1999).

According to Yahgan cosmogony, there was an ancient time in which the morphological differentiation between humans and other animals, plants, rocks or mountains was diffuse (Gusinde, 1986). Even the seasons did not originally exist. In ancestral times, trees never lost their leaves and were always green. However, one of their ancestors, the parrot *Kamshoat* or Austral parakeet (*Enicognathus ferrugineus* (S Müller, 1776)), began to paint the leaves giving rise to autumn. The trees revived months later giving rise to spring. This was the beginning of the annual cycles (Bridges, 1952, p. 455).

In the Yahgan worldview, humans form communities with other species. Ethical‐normative messages are communicated among humans and other species. Among recurrent themes is the radical questioning of selfish attitudes, such as hoarding common goods like food or water. For example, the transgression of misappropriating water is represented by *Cilawáia* a selfish fox (*Lycalopex culpaeus* subsp. *lycoides* (Philippi, 1896)) and the exemplary sanction for hoarding this vital element was death (Gusinde, 1961; Rozzi, Massardo, Anderson, et al., 2010).

### 2.2 Habits

**Figure 4:**
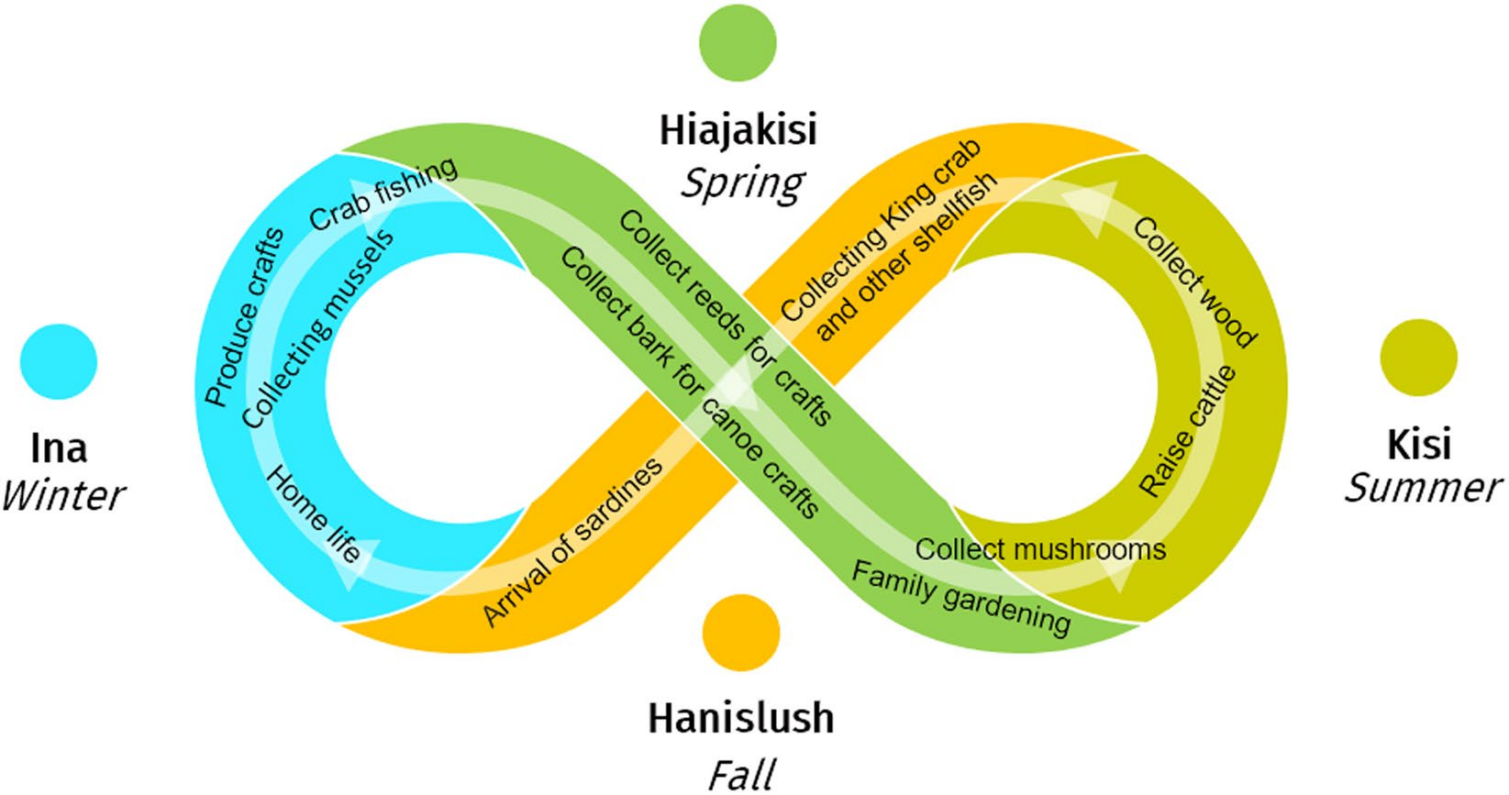
Yahgan biocultural calendar. The colors indicate the seasons of the year, and main cultural practices that in different seasons depend on specific *habitats* (e.g., marine‐coastal, wetlands or forests), characteristic life *habits* (e.g., fishing or gathering) and biocultural keystone *co‐inhabitants* (e.g., king crabs, mussels and other shellfish, rushes or fungi of the genus *Cyttaria* Berk., 1842, in *Nothofagus* forests).

Throughout the year, Yahgans use lunar cycles and their correspondence with tides as measures of time and reference to plan future actions; for example, to plan when a group should return or when to meet somewhere (Yesner, 2004). To fix meeting places over longer periods, seasonal phenomena are indicated; for example, the laying of eggs by wild geese (representing spring) or the emaciation of shellfish (representing winter) (Vallejos, 2009). During winter, activities are concentrated in the protected fjords and channels, where mussels and other shellfish provide basic sustenance (Figure 4). In spring most activities take place in the oceanic coasts exposed to the Pacific Ocean that are rich in fish and breeding areas for birds and marine mammals (Vallejos, 2009).

Spring and summer involve hunting birds and sea mammals, and gathering a variety of edible fungi and berries as well as tall rushes for basketry. Bark of the evergreen beech commonly called either Magellan's beech, coigüe or guindo (*Nothofagus betuloides* (Mirb.) Oerst.) is collected for the construction of canoes (Ballbe & Escalera, 2002). Fall and winter are the seasons for gathering sea‐urchins and mussels (Jackson & Popper, 1980). Today, seasonality is maintained for fishing activities that are regulated by the Chilean Undersecretary of Fisheries and Aquaculture (Rozzi et al., 2017). Seasonality also influences handcraft activities, such as basketry and construction of small canoes for special interest tourism (Massardo & Rozzi, 2006).

### 2.3 Co‐Inhabitant

Co‐inhabitants play central roles sequentially in different seasons of the year (Figure 4). In spring, epiphytic *Cyttaria* fungi develop their fruiting bodies or stroma (Palfner et al., 2022; Salazar‐Vidal, 2019). The genus *Cyttaria* has a long history of co‐evolution with *Nothofagus* trees, dating back to Gondwana about 100 million years ago (Swenson et al., 2001). This *Cyttaria*‐*Nothofagus* co‐evolution illustrates how ecological calendars have long‐term inter‐species relationships over broad temporal and spatial scales. In the Holocene, humans arrived in southern South America; thereby, ecological calendars became biocultural calendars. For example, *Cyttaria* fungi marked the beginning of spring and became a staple food providing carbohydrates and proteins for the Yahgan people (Schmeda‐Hirschmann et al., 1999).

In Spring and early summer, the bark of *Nothofagus betuloides* can be harvested to build canoes that enable the Yahgan people to navigate and inhabit the archipelagoes of Cape Horn (González et al., 2017). After the last glacial maximum, approximately 13,000 years ago, the archipelagoes emerged and were rapidly covered by evergreen forests as the ice *s* retreated (Davies et al., 2020). Using bark canoes, the first humans arrived at the Cape Horn archipelagoes at least 8,000 years ago (Orquera & Piana, 2020). The bark can only be effectively removed during Hakuerum, “the time when the bark is loose,” revealing a deep understanding of how tree sap behaves seasonally (Vallejos, 2009, p. 245). The bark extraction procedure is carried out on a single face of the trunk. In this way, the cutting of the sap flow by the phloem is avoided and the tree continues to live (González et al., 2017, Östlund et al., 2020). These culturally modified trees evoke similar practices in the Pacific Northwest (Mobley & Eldridge, 1992) and Scandinavia (Andersson et al., 2008; Östlund et al., 2020).

**Figure 5:**
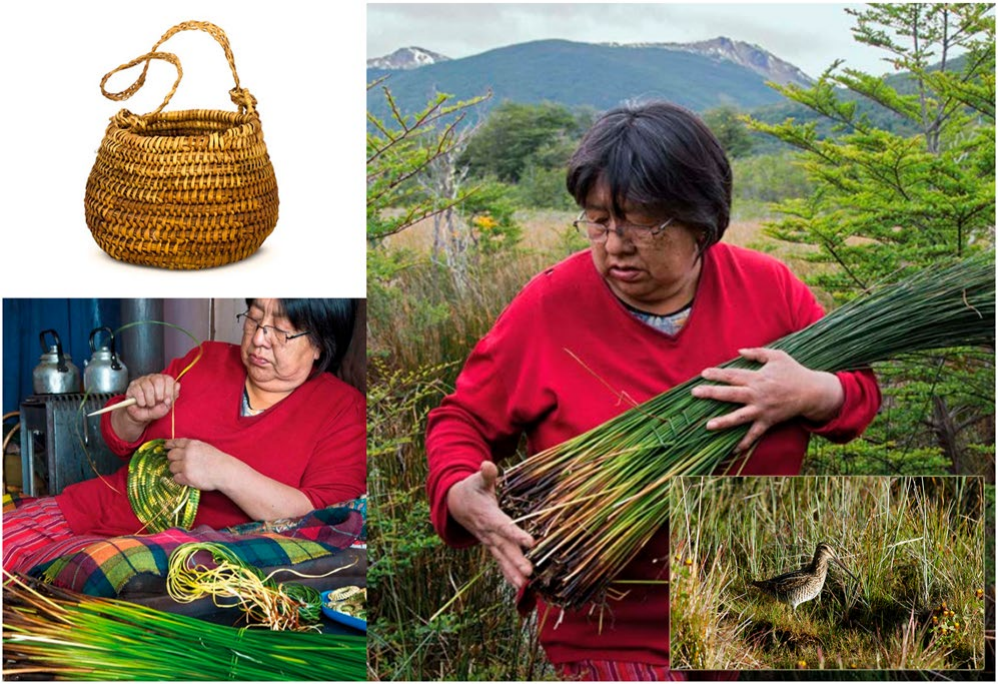
In the Cape Horn Biosphere Reserve, wetland habitats provide fibers needed for basket weaving as well as nesting and feeding sites for snipes (*Gallinago paraguaiae* subsp. *magellanica* (King, 1828)). Hence, humans and birds can be considered as co‐inhabitants in these habitats. Julia González, an artisan from the Yahgan Indigenous community, gathers *Marsipposerpum grandiflorum* rushes with which she weaves baskets. This image shows a *steapa* basket woven by Julia. Photographs by Jordi Plana (bird), Paola Vezzani (Julia weaving and gathering rushes), and Cristian Valle (basket). Figure modified from Rozzi et al. (2010).

In spring‐summer, another biocultural keystone plant species for the Yahgan people is the tall rush (*Marsippospermum grandiflorum* (L.fil.) Hook.fil.). Different types of baskets are made with its fibers: *steapa* with a tight weave are used to collect berries, and *keichi* with a loose weave are used to collect shellfish (Massardo & Rozzi, 2006). Groups of women collect rushes from wetlands, thereby stimulating other relational activities through collective basketry practices that, in turn, reinforce both social and inter‐species bonds through relationships of co‐inhabitation among humans, plants, and birds, such as snipes or *shakoa* (Figure 5).

Fall (*Hanislush*) is the season that begins when the leaves of the high‐deciduous beech or *hanis* (*Nothofagus pumilio* (Poepp. & Endl.) Krasser) turn red (*lush*). Fall is also marked by the arrival of abundant sardines (Figure 4). This phenomenon is called *Iacasi* when “seals, penguins, albatrosses and other seabirds, and deep‐sea fish, which arrived in fall chasing the school of sardines [… marking a] a time of superabundance for the Aborigines. The arrival of the *Iacasi* was celebrated with a harvest festival that could last up to 2 months” (Bridges, 1952, p. 75). In Fall, guanacos (*Lama guanicoe* (Müller, 1776)) and caiquenes (*Chloephaga picta* (Gmelin, 1789)) are fat, and can be hunted more easily (Bridges, 1952).

In winter, mussels (*Mytilus chilensis* (Hupé, 1854)) inhabiting the intertidal zones are central for the Yahgan diet, because they exhibit low seasonal variation in abundance (Ojeda et al., 2018). This constancy throughout the year was essential for human settlement (Jackson & Popper, 1980) by providing a constant food source supplemented with other species according to their temporal availability (Ojeda et al., 2018; Orquera & Piana, 2009). Gusinde (1986, p. 471) highlighted the importance of the mussels, pointing out that “the molluscs are for the Fuegian aborigines what bread is for the European.” Mussels represent a risk‐reducing factor that neutralized seasonal fluctuations in food supply (Orquera, 2000) and were part of the diet in rituals (Koppers, 1997). The banks of mussels were a determining factor for the choice of settlement sites (Orquera, 2000). These molluscs not only provided a food base, but their shells were essential for the construction of their huts or *akar*, where they were intentionally deposited to generate permeable substrates in very rainy areas (Verdún, 2010). The shell middens remain today as archeological sites found in practically all the archipelagos of the Cape Horn Biosphere Reserve (Álvarez et al., 2004; Ocampo & Rivas, 2000, 2004).

### 2.4 Conflicts

Today, the region of Cape Horn faces major conservation challenges because the “protective barriers” that have helped to preserve its pristine character are now being eroded. Its remoteness is being eliminated by rapid transportation and infrastructure development. New access roads being constructed through primeval forests in the Patagonian archipelago contribute to development projects that create negative environmental and social pressures (Barros & Harcha, 2004). For example, exponential growth of the tourism industry through cruise ships in areas previously restricted by the Chilean navy has led to an increasing number of tourists disembarking on uninhabited islands and to unregulated tourism in channels and protected areas. These lack basic infrastructure, tour‐guide information, and park rangers. Today, this type of unregulated tourism poses a threat to the most secluded spots in this remote wilderness region (García, 2004; Rozzi, Massardo, Cruz, et al., 2010). Additionally, exotic species are seriously impacting native terrestrial, freshwater, and marine biodiversity (Crego et al., 2015, 2018; Maldonado‐Márquez et al., 2020; Schüttler et al., 2019). In terrestrial ecosystems, a rapid spread of deliberately or accidentally introduced exotic vertebrate species, such as the American beaver (*Castor canadensis* (Kuhl, 1820)), American mink (*Neovison vison* (Schreber, 1777)), and muskrat (*Ondatra zibethicus* (Linnaeus, 1766)), is having a major impact on forest and wetland habitats and is decimating bird populations, especially of ground nesting species (Ibarra et al., 2009; Schüttler et al., 2009). In marine and freshwater ecosystems just north of the Cape Horn Biosphere Reserve, the rapid growth of industrial farming of nonnative salmon by using large numbers of floating cages anchored directly to the seabed is disrupting the austral sea and landscapes (40°–54°S) (Alvial, 2013). Salmon farming has major ecological and social impacts, including antibiotic pollution, eutrophication of lake and marine waters, introduction of voracious nonnative predator fish species, viral infections, and displacement of traditional fishing communities from their ancestral grounds (León‐Muñoz et al., 2007).

In the 19th century, the government of Chile promoted the European colonization of these lands, arguing that they were hostile, inhospitable and unproductive; however, they offered natural resources for their exploitation (Álvarez et al., 2017). In the process, populations of Indigenous peoples were brutally treated, expelled from their territories and seas, and even distortedly declared extinct in national historiography (McEwan et al., 2014). However, these communities are alive. During the last two decades programs have been established to recover and revalue their knowledge as well as the unique biodiversity of their archipelagic environments (Contador et al., 2018; González et al., 2017; Rozzi et al., 2006, 2010a; Zárraga et al., 2006). Today, members of the Yahgan community are revitalizing their language, and cultural practices that are coupled with the life cycles of animals and plants (Figure 4, Section 2.5).

### 2.5 Box 1 Biocultural Conservation and Intercultural Education in Cape Horn

**Figure 6:**
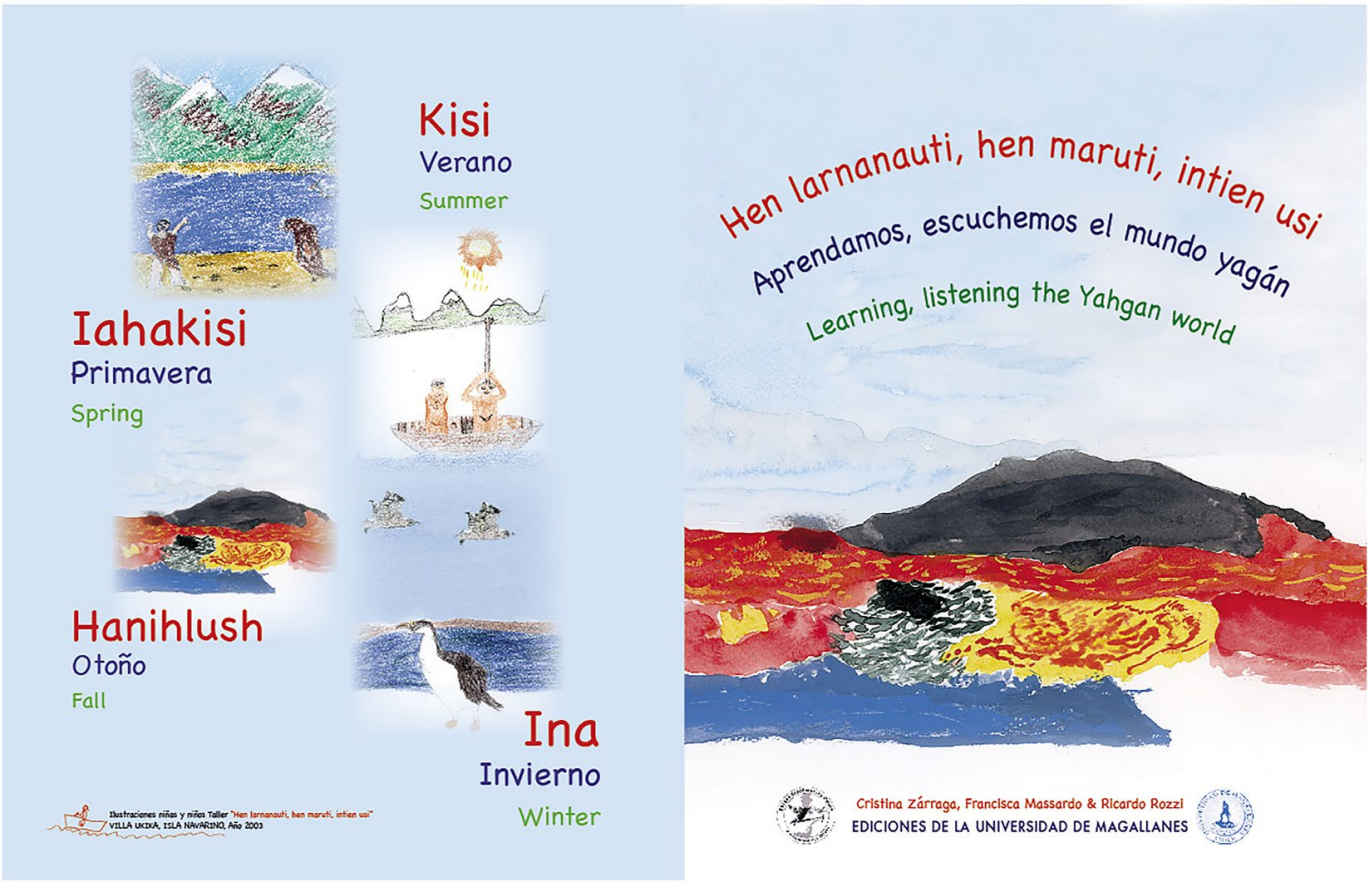
Cover of the Yahgan Dictionary *Hen larnanauti, hen maruti intien usi* (“Learning from and listening to the Yahgan world”) that includes illustration made by children. The cover illustrates the four seasons of the year illustrated by boys and girls of the workshop who understood the meaning of words and cultural activities linked to the life cycles of plants and animals of the sea, coasts, wetlands, and forests. The book includes a CD with the recordings of words in trilingual format, Yahgan‐Spanish‐English to foster intercultural education. Cover image from Zárraga et al. (2006).

To illustrate the biocultural conservation initiative in Cape Horn (Rozzi et al., 2006), we concisely present an intercultural education experience regarding the relationships between the Indigenous Yahgan language and the natural world in the archipelagoes of Cape Horn at the southern end of the Americas. In 2003, with elders, youngster, and children of the Yahgan community we conducted a participatory workshop with Grandmother Cristina Calderón who was the only person who still fluently spoke the Yahgan language at that time. She shared her memories and spoke the Yahgan language to the young people, evoking landscapes, languages, tools, and ways of inhabiting the seas and lands that ancestrally constituted their territories (Zárraga et al., 2006). The children understood how the seasons of the year were indicated by the rhythm of nature (Figure 6). As such, *hanislush*, autumn, is associated with the color red (*lush*) that is acquired from the high‐deciduous beech tree (*hanis*) on the slopes of the mountains (*tulara*) in the fall. *Ina*, winter, is marked by long nights and snow, associated with *lampia* (black) and *yakua* (white), respectively. *Iahakisi* (spring) the short (*iaha*) summer (*kisi*), is signaled by the shoots of leaves that color this season with *arlampia* (green). *Kisi,* the summer, arrives with its interminable days where *kurlampia* (blue) inundates the sea and sky.

This experience stimulated children to recover their Yahgan ecological knowledge about the seasons of the year, sky, sea, fauna, flora, landscapes, tools and life habits to coinhabit the islands, fjords and channels of Cape Horn. Their forms of co‐inhabitation with the natural world offer a biocultural orientation not only for the young people of the Yahgan community, but also for the youngsters of other regions of the world.

## 3 Biocultural Calendars in the Archipelagoes of Chiloe and Western Patagonia

### 3.1 Habitat

#### 3.1.1 Biophysical Habitat

Fisher communities inhabit the archipelagoes and fjords of southwestern Chile (Figure 2). The western margin of southern South America was shaped by the advance and retreat of glaciers in the Pleistocene (Silva & Calvete, 2002), generating an area of fjords with a steep coast and inland seas. Flows of matter and energy are concentrated in this inland sea and in the channels and fjords, reaching the highest values of marine productivity in the region (Pantoja et al., 2011). Due to the close terrestrial‐marine link in Chilean Patagonia and the profusion of fluvial courses, coastal ecosystem dynamics are influenced by continental ecological processes, including large tidal changes and dragging of sediments and organic matter from rivers to the sea (Pantoja et al., 2011). This is especially relevant in areas of marshes and coastal wetlands. The weather is oceanic and strongly influenced by the westerlies that bring humidity (Rozzi et al., 2000). Annual rainfall fluctuates between 3,000 and 4,000 m, and annual mean temperature is 9ºC.

#### 3.1.2 Symbolic Linguistic Habitat

A mestizo worldview prevails in the culture of the diverse communities that inhabit the Chiloe archipelagoes of southern Chile (Álvarez & Ther, 2016). Prior to European colonization, two Indigenous groups occupied these archipelagoes: the Chonos and the Williche (Daughters, 2018). Chonos people were nomadic and navigated in canoes carved from trunks. They relied on a diet of fish, shellfish, potatoes, and occasionally sea lions. Although the Chonos ceased to exist as a distinct ethnic group in the nineteenth century, aspects of their culture have persisted (Álvarez, 2012; Cárdenas et al., 1991). The Williche are sedentary but do some hunting, fishing, and gathering of seaweeds, fungi, and fruits. However, they rely on agriculture and their most important crop is the potato, a plant native to Chiloe that includes over a hundred varieties cultivated in the archipelago (Massardo & Anderson, 2001). A particular variety of potato is adopted by each newly married couple on the islands of Chiloe (Massardo & Anderson, 2001).

The southern winter solstice, between 21 and 24 June, marks the celebration of the “Mapuche New Year” or *wetripantu*. It is the shortest day of the year, and it marks the beginning of the retreat of winter. Hence, every day has increasingly longer length of sunshine, propitiating a new cycle of agriculture and the renewal of nature. The sequence of Mapuche farming and fishing activities has been described as a socio‐geolitoral calendar (Gajardo & Ther, 2011). The concept of socio‐geolitoral underlines the heterogeneity of the coast; that is, each bay is biologically and culturally unique (FSP, 2016, 2018). Each community alternates fishing activities with agricultural and forestry tasks according to the life cycles of marine and terrestrial species. In turn, these biological cycles are coupled to astronomical calendars; that is, the annual and monthly cycles of sun and the moon, respectively (Gajardo & Ther, 2011). This pluri‐activity yields monetary dividends, but it yields higher social and environmental dividends. It is resilient in the long term, since customs provide normative restrictions that morally sanction the possibilities of hoarding, selfishness, and other actions that are detrimental to the relationship between people and nature. In addition, multiple species and ecosystem components are part of the material and symbolic culture. They can be considered as co‐inhabitants beyond being just food, or materials to build something. In oral tradition central characters are often other‐than‐human co‐inhabitants who convey normative‐ethical values (Álvarez & Ther, 2016).

### 3.2 Habits

**Figure 7:**
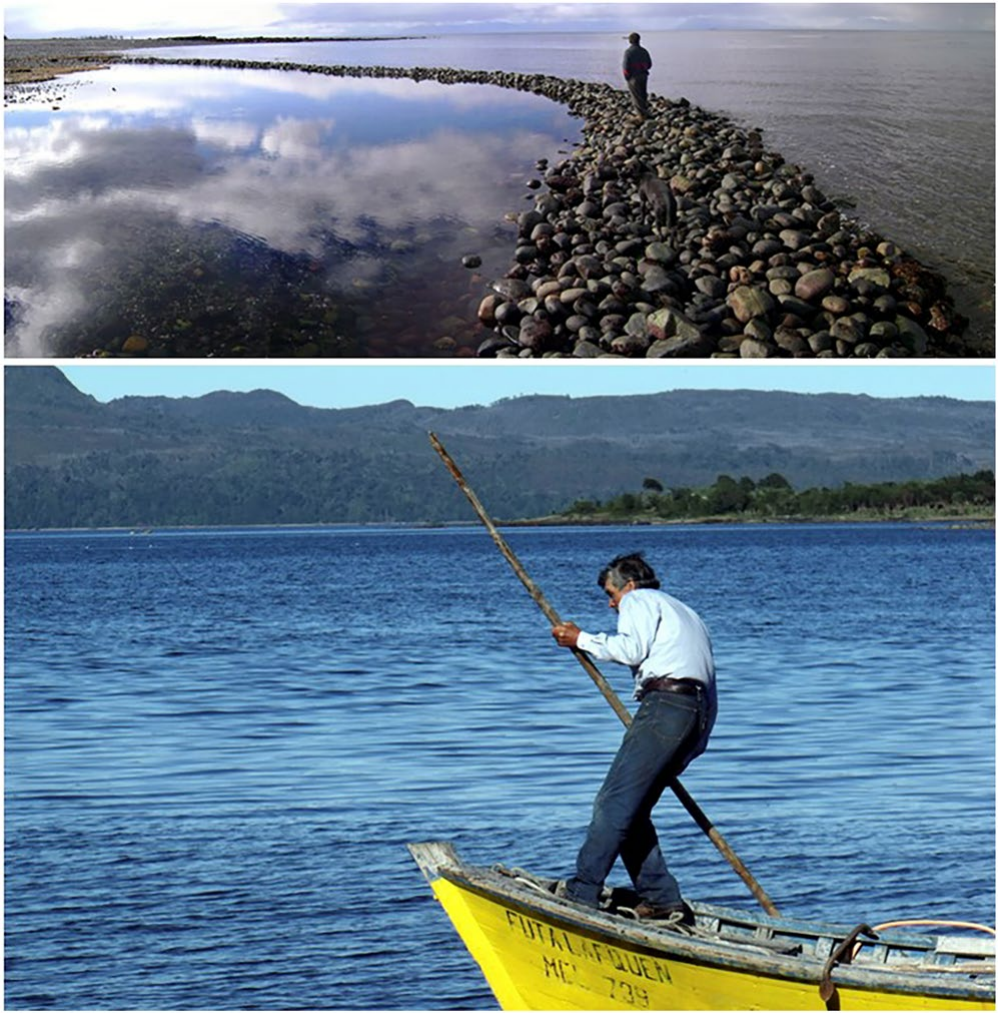
(top) A fishing weir on Caguach Island, Chiloe archipelago. This stone dam is traditionally built to catch fish taking advantage of the cycle of the tides, the moon, and the seasonal migrations of fish. (bottom). Local fisherman on his boat in the Guaitecas archipelago south of Chiloe (Photographs Ricardo Álvarez, 2008).

The relationship between cultural manifestations and natural cycles can be illustrated by the practice of the fishing weir, stone or wooden “corrals” that serve to capture fish taking advantage of the dynamics of the tides (Figure 7). Today, fish weirs are also used as storage sites for algae and other functions. In Chiloe there are a thousand of these “corrals” that were used by island families until recent historical times (Álvarez, 2016; Álvarez et al., 2008).

**Figure 8:**
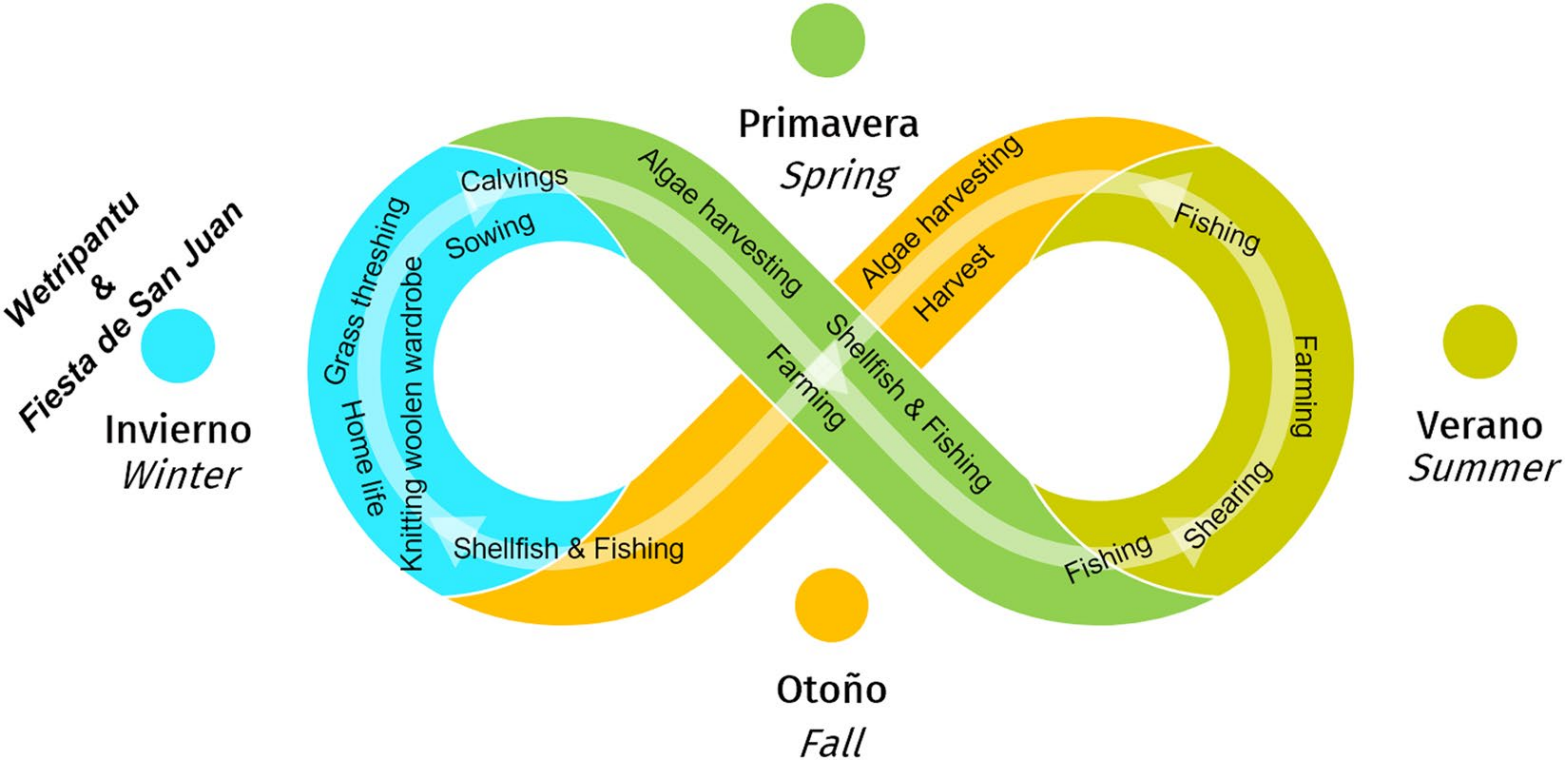
Biocultural calendar in the Chiloe archipelagoes. Seasonally, these practices depend on specific *habitats* (e.g., marine‐coastal, pasturelands), characteristic *habits* (e.g., algae harvesting, fishing, potato planting, or sheep shearing), and *co‐inhabitants* that can be considered biocultural keystone species, such as red algae like *Gracilaria* spp. (Greville, 1830) and *Sarcothalia crispate* (Bory) Leister, Patagonian blennie (*Eleginops maclovinus* (Cuvier, 1830)), and other fish, potatoes, and sheep. Family work also includes elaboration of handicrafts based on elements gathered in marine‐coastal and forest habitats. Two festivals are celebrated during the winter solstice: *wetripantu* of Indigenous origin, and the *Fiesta de San Juan* of Hispanic origin.

In the islands of Chiloe, an oral memory persists that maintains active biocultural calendars (Figure 8). This customary island model is especially vital on smaller islands (<80 km^2^) inhabited by ca. 12,000 people (FSP, 2018; Skewes et al., 2012) who host intertwined belief systems derived from historical miscegenation. For example, potatoes and other tubers are still planted with a waning moon due to its cosmogonic equivalence with the underground (Urriola, 2018). In contrast, species that grow toward the sun, such as wheat, should be planted with a crescent moon. The full moon and new moon are associated with very intense tides or “pilcanes,” which are ideal for marine‐coastal work, such as shore harvesting. In contrast, during the waning moon phase, agricultural work carried out by families in the interior habitats of the islands is privileged. However, the activities in these contrasting periods and habitats are interrelated. For example, the cultivation of potatoes and other crops requires seaweeds as a natural fertilizer (Cardenas et al., 1991). Sea lettuce (*Ulva* sp. (Linnaeus, 1753)) and other seaweeds are harvested during the pilcanes (Álvarez et al., 2008). In short, in Chiloe, agriculture, fishing, and gathering practices are interconnected in complex lunar, biological, and cultural calendars (FSP, 2016).

### 3.3 Co‐Inhabitants

*Williche* people synchronize the use of fishing weirs with cycles in which biocultural keystone species of fish such as jack mackerels or “jurel” (*Trachurus murphyi* (Nichols, 1920)) and snoeks or “sierra” (*Thyrsites atun* (Euphrasen, 1791)). During their spawning period, these large fish approach the coast chasing schools of sardines. Fishing weirs are used throughout the year to capture other species such as the Patagonian blennie or “róbalo” (*Eleginops maclovinus*). Finally, fishing weirs are a refuge for multiple intertidal species, such as rock fish, crustaceans, and seaweeds that support coastal families. Thus, fishing weirs generate relationships of human‐nature reciprocity since these anthropogenic habitats stimulate greater biodiversity at the beaches (Sepúlveda, 2017). This type of reciprocity extends beyond fishing weirs to inter‐tidal and sub‐tidal micro‐ecosystems that are protected by local fishers to stimulate the proliferation of multiple species, including seed tunicates called “piures” (*Pyura chilensis* (Molina, 1782)) and carnivorous mollusks, such as the Chilean abalone or “loco” (*Concholepas concholepas* (Bruguière, 1789)). In 1989, these fishing habitats and habits were included in the Chilean legal decree 18,892 on Benthic Resources Management and Exploitation Areas.

### 3.4 Conflicts

Since mid‐twentieth century, neoliberal economic policies have instilled private property rights, economic efficiency, and deregulation (Pinkerton, 2017). This has impacted local fisheries in terms of access to coastal habitats and loss of traditional life habits. National policies have overridden biocultural calendars by imposing fishing calendars governed by markets. However, local habits, knowledge, and regulations are still present in fisher communities, and these maintain synchronization with biocultural calendars (Gajardo & Ther, 2011).

Fishing activities conducted in fish weirs and small boats along the coasts (Figure 7) are in marked contrast with mono‐species dependency driven by the market. The latter imposes marked, discrete periods of fishing activity with legally established closed seasons. Market calendars treat fish, other species and marine ecosystems as mere natural resources. The establishment of industrial extractive methodologies and neoliberal fishing regulations is spoiling seasonal practices of fishing weirs, and contributing to decreasing schools of fish.

The aquaculture industry, especially salmon farming, is another pressure that has triggered the decoupling of productive activities and biocultural calendars. Since the 1980s, salmon farming has had cultural, social, and biophysical consequences such as harmful algal blooms that impact the economies of thousands of local fishing families and affect native species (Armijo et al., 2020). Salmon farming has also precipitated the breakdown of relationships between fishers and marine ecosystems and species, including marine mammals (Bedriñana‐Romano et al., 2021). These mammal species were part of the Indigenous diet and worldviews. However, today industrial aquaculture considers otariids and cetaceans threats to salmon farming cages and efforts are made to eliminate them from coastal waters (Osman et al., 2007).

Complex mestizo cultures (generated by the coexistence of industrial practices and worldviews with Indigenous habits and worldviews) offer opportunities for adapting to current social‐environmental problems. For example, today local fishers recognize the high intelligence of sea lions and maintain a “mutual understanding” of co‐inhabitation. Fishers must vary their fishing practices to avoid interference by sea lions which quickly detect the new sites, baits, and techniques used by fishers (FSP, 2018). On the other hand, while navigating in boats to set or collect catches, fishers and sea lions learn from each other how to detect schools of fish thereby establishing genuine inter‐species relationships (FSP, 2016). These forms of interspecies co‐inhabitation reveal a common language between human and other‐than‐human co‐inhabitants. This inter‐species language is sensitive to annual cycles. Both sea lions and humans experience times of abundance and cycles of scarcity.

## 4 Lafkenche‐Williche Biocultural Calendar

### 4.1 Habitats

#### 4.1.1 Biophysical Habitat

Coastal forests, shrublands, pasturelands, and marine habitats in southwestern Chile, between 35°S and 41°S, are inhabited by Lafkenche‐Mapuche communities that gather algae, mussels, and fish. In the Indigenous language Mapudungun, *Lafkenche* means people (*che*) of the sea (*lafken*) (Massardo & Rozzi, 2014). Evergreen rainforests and coastal ecosystems between 38°S and 43°S are inhabited by Williche‐Mapuche communities. In Mapudungun, *Williche* means people of the south (*willi*) (Massardo & Rozzi, 2014). The climate is humid with an annual rainfall that varies between 2,000 mm (near the coast) and 1,000 mm (inland sites) (Ramírez‐Martínez, 2008), and annual mean temperature is 13.5°C (Espinoza et al., 2003). This seasonality is characteristically Mediterranean with three rainy seasons, and warmer and less rainy summers (Errázuriz‐Körner et al., 1999).

#### 4.1.2 Symbolic Linguistic Habitat

The Lafkenche and Williche are Mapuche communities that have historically inhabited the *Lafkenmapu,* or coastal (*lafken*) land (*mapu*). For them, this territory has both material and symbolic cultural significance (Pardo‐Pérez, 2016). The spatial and temporal dimensions of the Lafkenmapu are organized on the basis of tetrads (Aillapan & Rozzi, 2014). Regarding the temporal dimension, the number four marks not only the seasons of the year but also the parts of the day. Lafkenche poet, Lorenzo Aillapan, expresses this view in his poem about *Tiftifken* or “Pocket‐watch Bird” (*Scytalopus magellanicus* (Gmelin, 1789)):

Millenary bird that works by the light of day, and this it divides into four parts: the daybreak, the morning, the noon‐time, and the afternoon; symbol of life, work, results and production, family, planting, harvest and animals (Aillapan & Rozzi, 2001, p. 17).

**Figure 9:**
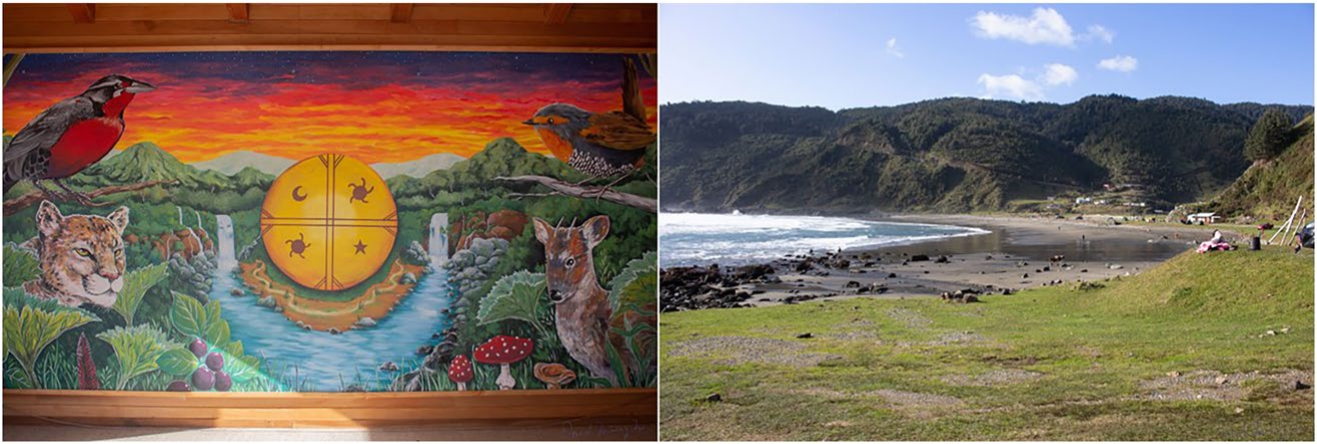
(Left) Mural painting at *Rokura* or Caleta Bonifacio (39º40’S) in the Lafkenche–Williche territory. At the center, the mural illustrates the Mapuche drum or *kultrun* depicting the four spirits linked to the cardinal points of the land. On the upper left side of the mural is the *Lloyka* or “healer bird” with its red chest. The mural shows forest, freshwater, and coastal habitats that are integrated in the Lafkenmapu. (Right) *Pilolkura* or Pilolcura Beach, adjacent to Rokura, where seaweeds and shellfish are gathered. Photographs by David Núñez.

With its calls, *Titifken* communicates to humans the rhythm of nature, harmonizing their daily and seasonal activities. In the Mapuche culture, the natural, human, and divine worlds live together, and birds are messengers across these worlds (Rozzi, 2014). Among birds, the *Lloyka* or Long‐tailed Meadowlark (*Sturnella loyca* (Molina, 1782)) possesses special powers to connect the human with the divine and nature (Figure 9). For example, this bird links the four cardinal points of the spatial dimension of the Lafkenmapu with four central divinities, the “Four Wind‐Spirits of the Earth:” from the west Dumpall, the guardian of the sea; from the southeast *Pillan*, the guardian of the volcanoes; from the northeast *Anchümallen,* the princess of the sun; from the north *Witranalwe*, the great wealthy and powerful visitor (see Faron, 1968). These four spirits and cardinal points are represented in the main percussion instrument of the Mapuche, the *kultrun* (Grebe, 1978) (Figure 9).

The *Lloyka* also connects four worlds along a vertical axis of the spatial dimensions of the universe.

1. *Wenumapu*, the upper (*wenu*) land, that is like a reflection of the *Mapu*; it is inhabited not only by planets and stars, but also by relatives and friends who have died and now are working there just like they did in the Mapu (e.g., farming, taking care of animals, conducting ceremonies) (Huaiquinao, 2003).
2. *Ankawenu,* is the sidereal space where birds fly and where wind, air, clouds, rain, and thunder dwell, which is the space between (*anka*) the *Wenumapu* and the *Mapu.*
3. *Mapu*, the land “of here” where human beings live, suffer, and love. *Mapu*, also called mother earth (*ñukemapu*), is delicate and must be loved. The land is worked freely and fraternally, like brothers (*peñi*). In the *ñukemapu*, birds and trees should be treated as equals. To achieve this, the ancestors consumed only what they needed.
4. *Minchemapu*, the land of below (*minche*) that encompasses all that is underneath, the rivers and the soil, is like the subsoil (Aillapan & Rozzi, 2014). This layer of water inside the earth is connected to lakes, and each lake is connected to the ocean through a *Ñe* or sea eye (Millaman‐Reinao, 2008).

In summary, in the Mapuche worldview, the temporal rhythms of nature are organized in four parts at annual and daily scales (Aillapan & Rozzi, 2014), and the spatial horizontal and vertical dimensions are organized in four circular spaces in the universe (Millaman‐Reinao, 2008).

### 4.2 Habits

The Mapuche culture stands out for its natural medicine based in native plants (Aldunate, 1996; Foerster & Gundermann, 1996; Massardo, 1997; Massardo & Rozzi, 1996; San Martín, 1983; Villagrán, 1998). In the 18th century, the Jesuit naturalist Juan Ignacio Molina admired the habits of Mapuche herbalists who gathered plants and prepared different infusions, and wrote that:

Vegetables, especially herbs, form the capital of the Mapuche pharmacy… and their doctors, called *machi* and *ampife*, are expert herbalists that possess, through tradition, the secret of a large number of simple [plants], adaptable to every kind of illness, with which they make marvelous cures every time; and although… they try to hide what they know in this matter, nevertheless, moved by friendship, they have demonstrated until today the medical virtues of many trees and more than 200 healing herbs that they use with much success (Molina, 1978, p. 117).

Today, Aillapan expresses how, just as the Mapuche shaman (*machi*), the *Lloyka* is a “full‐time healer bird” that enhances the growth of medicinal plants. In Aillapan's poem the bird says: “I have always healed my people with pure herbs. For this, my name is full‐time healer” (Aillapan & Rozzi, 2014, p. 325). The Mapudungun name lloyka stems from the terms *llako* (healing) and *lawen* (remedy). Aillapan explains that this healer bird wishes to cure its own gaudy red colored chest that carries the blood of the Mapuche people (Figure 9).

In the forest, not only medicinal plants are collected, but also mushrooms, edible plants and animals, firewood for cooking and heating houses, materials for building houses and making utensils for domestic and agricultural uses (Barreau et al., 2016; Ward, 2003). In basketry, climbing plants or *voqui* are essential. Lafkenche and Williche people use a variety of *voqui* such as *Asteranthera ovata* (Cav.) Hanst. (Gesneriaceae), *Cissus striata* Ruiz & Pav. (Vitaceae), *Eccremocarpus scaber* Ruiz & Pav. (Bignoniaceae), *Elytropus chilensis* (A.DC.) Müll.Arg. (Apocynaceae), *Lapageria rosea* Ruiz & Pav. (Philesiaceae), *Tropaeolum speciosum* Poepp. & Endl. (Tropaeolaceae), *Lardizabala funaria* (Molina) Looser, *Boquila trifoliolata* (DC.) Decne. (Lardizabalaceae), and *Berberidopsis corallina* Hook.fil. (Berberidopsidaceae) (Christenhusz, 2012). The latter is a monotypic endemic climber highly threatened by the replacement of native forest by plantations of eucalyptus and Monterrey pine (Armesto et al., 2001). The habits of gathering products are linked to handcraft practices and social customs that transform forests into highly valued habitats.

Along the coasts, gathering activities also take place in intertidal and subtidal marine habitats that host a variety of seaweeds, fish, and shellfish (Vargas‐Fernandez, 2021). Harvesting seaweeds, such as “cochayuyo” or southern bull kelp (*Durvillaea antarctica* (Chamisso) Hariot, 1892), involves synchronizing harvesting and drying habits with the periods in which the seaweeds grow. In addition, the climate that allows them to be dried on the shore for storage and marketing is considered; otherwise, it would rot.

In the Lafkenche territory, cultural practices are synchronized with natural cycles in common lands where gathering habits are linked to an ethics of solidarity. The accumulated experience in periods of seasonal scarcity, mainly during winter, stimulates solidarity‐type cultural practices (Araos et al., 2019). Food shortages are solved through joint work to establish food security thresholds that are common among families: no one should have neither more nor less than anyone else. Those who are left behind (*kuñifal*) are immediately attended to because their precariousness causes social imbalance (Álvarez et al., 2018). Solidarity extends beyond humans, supporting a biocultural ethic spanning to all species with which human communities coinhabit.

In summary, according to the Mapuche worldview, human and other‐than‐human beings can be understood as co‐inhabitants who strive as a biocultural community for well‐being (Rozzi, 2018). This biocultural community favors the regeneration of life when it is altered (Skewes, 2019). In turn, this regenerative capacity might be particularly relevant to confront climate change, and it underlines the necessity to conserve both biological and cultural diversity in the Lafkenmapu.

### 4.3 Co‐Inhabitants

**Figure 10:**
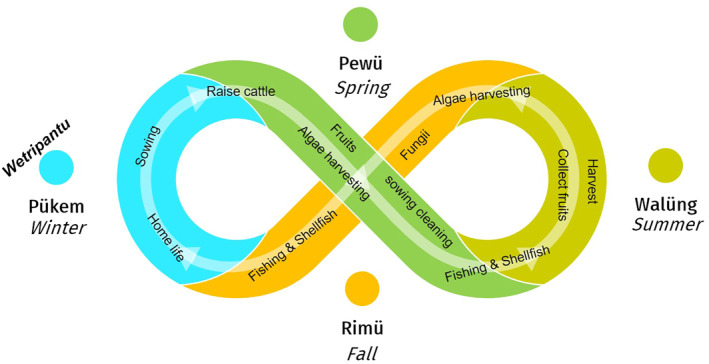
Lafkenche‐Williche biocultural calendar. In different seasons, cultural practices depend on specific *habitats* (e.g., marine‐coastal, grasslands, or homes), *habits* (e.g., seaweed or shellfish harvesting, grazing, or handicraft making), and *co‐inhabitants* (e.g., seaweed, shellfish, or livestock) who are biocultural keystone species. At the winter solstice, the *wetripantu* marks the beginning of the Mapuche new year, involving ritual activities.

Different co‐inhabitants are central in different seasons of the year (Figure 10). In fall (*rimü*) a variety of fungi are gathered. Community activities are reduced giving place to conversations within each family, usually commenting on the experiences they had during the spring and summer. In winter (*pükem*), the season of greatest deprivation, community practices include times for collective thought and reflection (Grebe, 1987). Spring (*pewü*) implies flowing with the vitality of plants and animals through community activities linked to respectful use of forests (Skewes, 2019), cultivation of polyculture gardens (*tukun*) inserted in a matrix of forests and shrublands (Muñoz‐Sáez et al., 2019), raising cattle on common lands, and collecting seaweed and shellfish on intertidal and subtidal habitats. On land, livestock and minor agriculture occurs in co‐inhabitation with birds and other native species along with post‐Columbian domestic animals such as cats, dogs, and pigs. At home, families transform the wool of their sheep into beautiful fabrics dyed with native plants.

In subtidal habitats, the clam *Ameghinomya antiqua* (P.P. King, 1832), the sea urchin *Loxechinus albus* (Molina, 1782), the sea snail *Tegula atra* (Lesson, 1830), and the crab *Taliepus dentatus* (H. Milne Edwards, 1834) are four keystone species for the Lafkenche diet (Vargas‐Fernandez, 2021). In the forest and shrublands, birds (such as the *Lloyka*) and plants (such as *voqui*) are salient (see above). Among human families, when faced with unforeseen events, such as periods of drought during spring or summer, Lafkenche and Williche families do not hesitate to exchange seeds that have been culturally modified for centuries and that they know will germinate and grow under stressful conditions (Montalba et al., 2015). In summer (*walüng*) they continue exchanging seeds and begin harvesting fruits. Additionally, young lambs and calves are born, thereby generating a season of opulence. Hence, ecological seasonal patterns and the sequence of social activities among Lafkenche people are closely linked. These biocultural links reinforce knowledge and customs that nourish a sense community, which includes human and other‐than‐human co‐inhabitants (Figure 10).

### 4.4 Conflicts

Since the Spanish arrival in Chile in 1537, the Mapuche territories have experienced three main waves of transformation (Rozzi et al., 2000). First, occupation by the Spaniards during the 17th and 18th centuries brought the spread of virulent epidemics, direct slaughter of Indigenous people, introduction of horses and cattle, and an increase in the extraction rates of forest trees for construction, furniture, and fuelwood (Bengoa, 1985; Donoso & Lara, 1996).

The second wave began after Chile gained independence from Spain in the 19th century. The Chilean government organized massive campaigns promoting the immigration of European colonists, especially German farmers. With the goal to “develop” the country (see Pérez‐Rosales, 1882), between 1860 and 1900 German immigrants burned great expanses of forest in Chile's lake region (39°–42°S), opening land for agriculture and cattle (see Ilg, 1982; Schmalz, 1970, 1971, 1972). As was happening simultaneously across the Andes in Argentinean Patagonia, semi‐nomadic peoples were displaced onto marginal reservation lands while the most productive regions of their former territories were granted to colonists from other regions of Chile, or to German, Italian and Swiss immigrants (Aylwin, 1994). Within two or three decades, the colonists had developed extensive monocultures of wheat whose consequential landscape degradation, including erosion problems, persist today (Donoso & Lara, 1996).

The third wave began in 1973 with the military coup (Solimano, 2014). Native forests began to be subject to accelerated processes of replacement by monocultures of *Pinus radiata* D.Don (Monterrey pine) and *Eucalyptus* L'Hér. species (Echeverria et al., 2006). Since 1974, these monotypic plantations with exotic fast‐growing tree species have been subsidized by Government Decree Law 701 that provides 75%–90% of the cost of planting and gives tax exemptions for the products obtained from these plantations (Pastor‐ Barrué, 2004). The massive substitution of native forests by exotic monocultures causes severe losses of biodiversity, soil erosion and compactness, and is detrimental to the hydrological cycle, provoking winter floods and summer droughts (Salas et al., 2016). Land‐use changes have promoted a strong migration of the rural population to urban centers for four main reasons:

1. Small owners have sold their lands to forestry companies; consequently, the concentration of the land ownership has displaced local communities.
2. Decrease in demand for labor, because forestry monocultures require less labor than traditional agriculture, and labor is required only during intermittent years for planting, thinning, and cutting.
3. Forestry companies bring their own labor force from other regions of Chile or the world; hence, this new economic activity provides few job opportunities for local people.
4. Native biodiversity is drastically reduced; consequently, multiple uses and values of native forests are eliminated.

The combination of these factors has caused a severe decline in the living conditions of Mapuche Indigenous communities (Andrade, 2019). Today, Mapuche resistance movements aim to stop the transformation of native forests into homogenous plantations of exotic trees (Reinao, 2008). They demand recovering access to the land, restoring native forest and its natural water cycles that have been disrupted significantly by monocultures (Torres‐Salinas et al., 2016, 2022). The Mapuche movement shows a great heterogeneity and includes different forms of political action from armed struggle to the promotion of a non‐violent solidarity economy (Schmalz et al., 2022).

The non‐violent or peaceful solidarity economy movement is based on deep cultural traditions. It centers on recovering ancestral lands, stopping the advance of environmental degradation, and reversing water scarcity associated with forestry monoculture by questioning the one‐dimensional capitalist logic of exploiting “natural resources,” and by reintroducing more sustainable ways of managing natural commons valued as communities of co‐inhabitants. This form of the Mapuche environmental justice movement offers an example to the world about how to supersede biocultural homogenization based on monocultures to instead foster biocultural conservation (Rozzi, 2018). On one hand, this movement stresses the relevance of deconstructing concepts of the coloniality of nature as well as historical, political, spatial, and socio‐environmental processes associated with mere production of commodities. On the other hand, the Mapuche worldview offers relational concepts, communities, and territories that orient decolonization struggles to restore lands and ancestral ontologies, which support the human right of coinhabiting the world in different ways.

The Spanish colonization and the subsequent establishment of the Republic of Chile also curtailed the access of Lafkenche‐Mapuche communities to coastal marine habitats. This was aggravated in 1991 with the promulgation of the General Law of Fishing and Fish Farming (Law 18.892), which did not acknowledge the presence of the Indigenous peoples on the coasts of Chile. This lack of recognition triggered a social movement by the Mapuche‐Lafkenche Indigenous people, producing a shift of the Indigenous demands from the interior lands to the sea. After over a decade of negotiations, in 2008, the government of Chile introduced the “Lafkenche Law” (Law 20.249) establishing a new conservation category in Chile: the coastal‐marine space of the original peoples (ECMPO, Espacio Costero Marino de Pueblos Originarios) (González‐Poblete et al., 2020). This law recognized for the first time in the history of Chile the Indigenous peoples' condition as inhabitants of the coast, which includes their rights to traditional uses (e.g., fishing, spiritual, recreational) in marine‐coastal areas (Molina‐Camacho et al., 2021). Nevertheless, agreement between the Chilean State and Mapuche‐Lafkenche communities has been very difficult to implement, leading to feelings of frustration and movements of rebellion (Gissi et al., 2017).

In summary, the current conflicts in the Lafkenmapu represent a serious threat to their ability to adapt to climate change. To promote resilience, it is essential to preserve the integrity of native habitats and access to them by the Lafkenche and Williche communities. Some existing public policies in Chile, such as the Lafkenche law, contribute to this, but it is necessary to enhance their implementation. This will allow not only conserving the biodiversity of the terrestrial and marine ecosystems, but also Mapuche cultural habits that are closely linked to other‐than‐human co‐inhabitants in the Lafkenmapu.

## 5 Aymara Biocultural Calendar in Northern Chile

### 5.1 Habitats

#### 5.1.1 Biophysical Habitat

**Figure 11:**
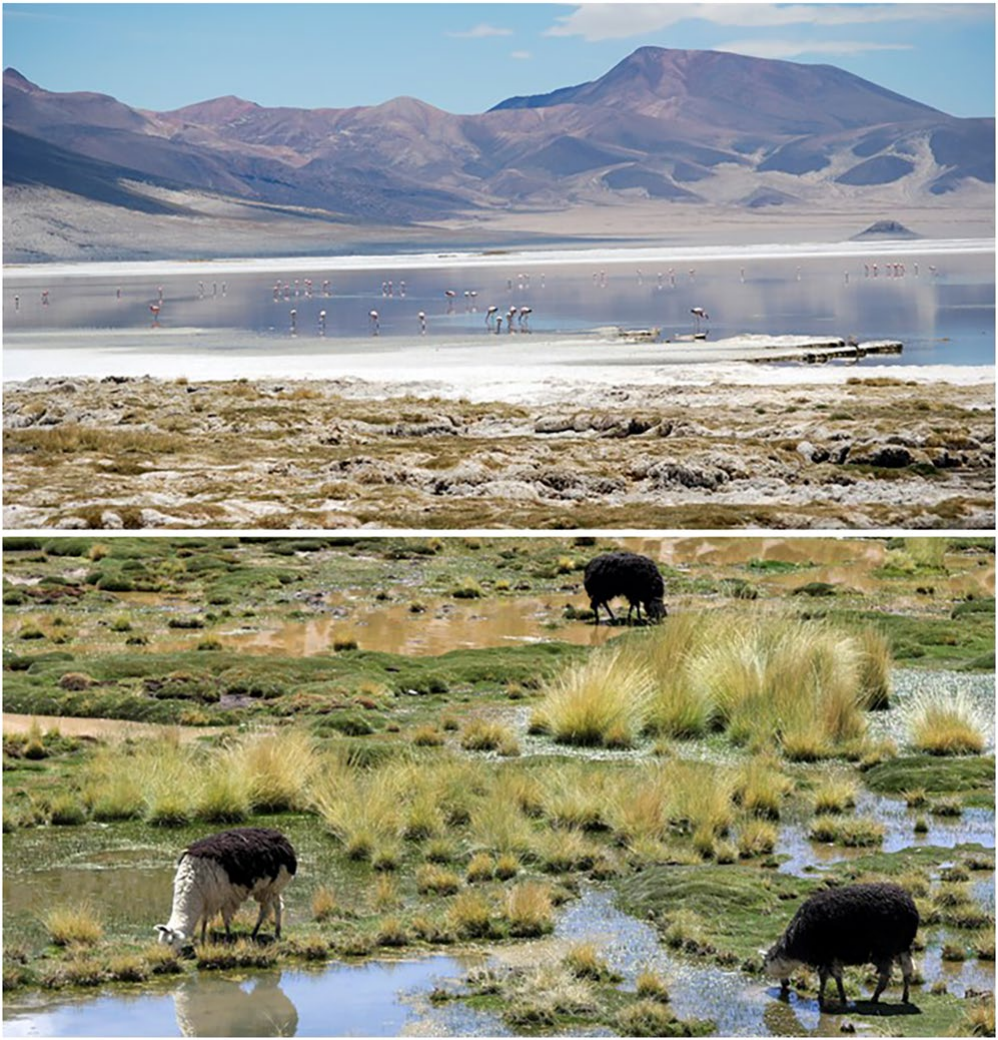
(a) Salar de Huasco, a salt flat dotted with ponds and salt marshes. It is seasonally flooded providing habitat for a rich diversity of bacteria, small invertebrates, algae, and vertebrates that include camelids and one of the world's largest populations of the Andean flamingo (*Phoenicoparrus andinus* (Philippi, 1854)) as well as Chilean flamingo (*Phoenicopterus chilensis* (Molina, 1782)). This habitat is particularly sensitive to changes in rainfall regimes that inform Aymara biocultural calendars (Photograph by Antonio Maldonado, 2011). (b) Pampas Lirima, a high Andean wetland that provides a key biocultural habitat for domestic camelids such as alpaca (*Vicugna pacos* (Linnaeus, 1758)) and llama (*Lama glama* (Linnaeus, 1758)) two species of South American camelids domesticated in pre‐Columbian times. Alpaca and llama are kept in herds that graze in the high Andes and are bred by Andean cultures for their fiber, and meat. Llama are also used as pack animals. (Photograph by Ximena Moreno, 2010).

A notable case of a biocultural calendar is found in the Salar de Huasco and Pampas de Lirima (20°N, 69°W) on the northeast border of Chile with Bolivia at 4,000 m altitude. This area has large wetlands supported by underground saline aquifers that are home to a great diversity of native fauna such as flamingos and camelids (e.g., vicuñas, alpacas, and llamas) (Figure 11). Summer rains characterize the climatic regime in the region and configure habitats that have been used ancestrally for agropastoral uses related to changes in rainfall (Moreno, 2011). The high Andean wetlands can be considered as “islands” within the desert region for pastoral activities. Combining ecological and ethnoecological methodologies, rainfall patterns have been identified at different altitudinal and ecological levels where pastoral activities are carried out allowing the conservation of biodiversity, phytomass, and functioning of ecosystems (Moreno, 2011). The region has two types of climates. First, climate of Tundra or of High Mountain Steppe that is associated with high altitude, dry winters with extremely low temperatures (−20°C), and summers that have abundant rainfall (400 mm) (DGA, 2009; Johnson et al., 2010). Second, cold desert climate influencing lower altitude zones with less extreme temperatures, smaller daily thermal oscillations, and moderate summer rainfall (150 mm) (Hernández‐López et al., 2014).

#### 5.1.2 Symbolic Linguistic Habitat

**Figure 12:**
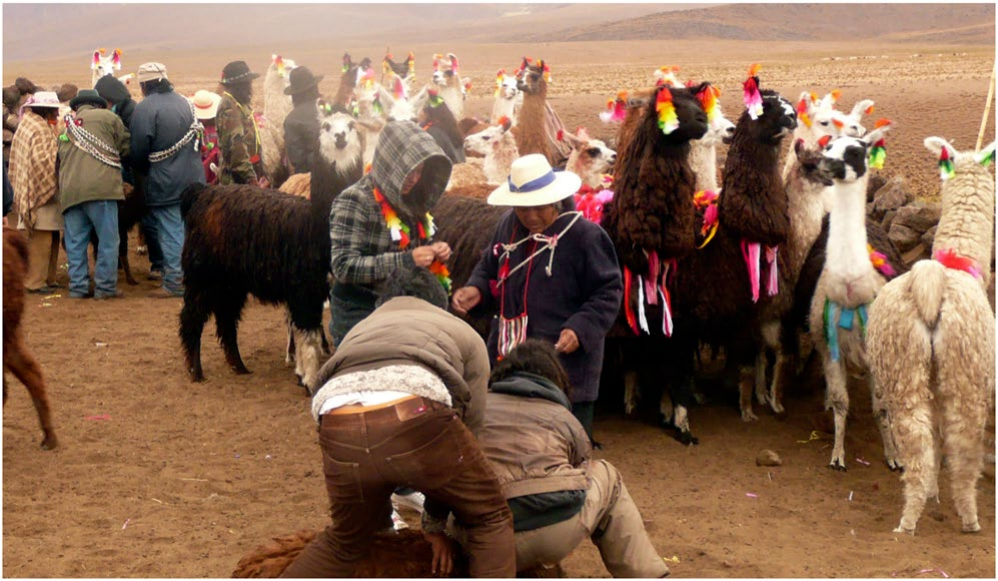
“Floreo” or marking of llamas in the Lirima pampas. Photograph: Ximena Moreno, 2008.

**Figure 13:**
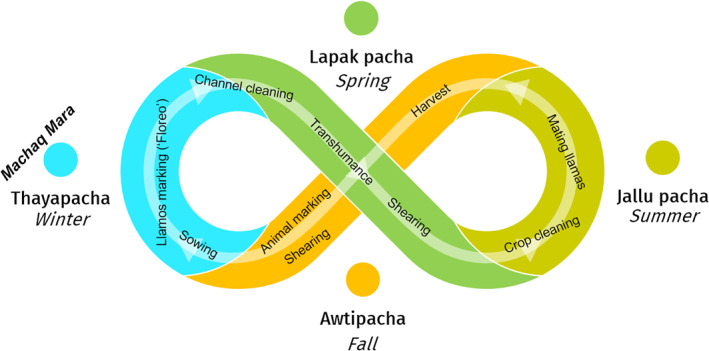
Aymara biocultural calendar. In different seasons of the year, main cultural practices depend on specific *habitats* (e.g., high Andean wetlands), *habits* (e.g., transhumant grazing), and *co‐inhabitants* (e.g., llamas, alpacas) who are biocultural keystone species. This calendar revolves around the breeding of camelids, but also integrates livestock of European origin, mainly sheep, aiming for a balance calculated on the availability of water in the wetlands. Aymara festive cultural practices stand out, including the “floreo” or marking of llamas in winter, and the *Machaq mara* celebration of the new year at the winter's solstice.

Andean worldviews entail social practices that involve human and other‐than‐human beings in the co‐construction of biocultural landscapes as well as economic and religious practices (Mamani‐Bernabé, 2015; May 2015, 2017) (Figure 12). High Andean communities have inherited an ancestral way to understand their environment, where the world is alive and inhabited by positive and negative forces. For example, mountains (*achachila*) are ancestors and sacred sites. These sites make the reproduction of life possible and must be respected and remembered in rituals and offerings. Their seasonal changes are the changes of human time; this is how Aymara biocultural calendars have been forged (Figure 13).

*Pacha* refers to both space and time (Mamani‐Bernabé, 2015). *Alax Pacha* is the world above; the abode of the sun and the moon; it is what is ahead in time and where the gaze is directed toward the future time (*Jutir Pacha*, a time, i.e., only known to the *Pachamama*, the material manifestation of *Pacha* as earth or Mother Earth). *Manqha Pacha* is the world below; it is the place under the Earth where what has already happened is kept, where the gaze is directed toward the past time (*Nayra Pacha*). It is a sacred space where spirits dwell, and where the waters are lost and the vegetation ends. *Aka Pacha* is at the center and harmonizes opposites; it is where the Aymara people live. It is where the present time (*Jicha Pacha*), in which we humans live, work, rest, and dream (Mamani‐Bernabé, 2015).

This symbolic base offers new combinations in the task of thinking about the projection of humans in the global order (Valdivia, 2006). The need to cooperate in the development of a sustainable planet finds in Aymara thought two important conceptions: the cult of Mother Earth or Pachamama as a “meeting point” and the right to “live well” in harmony with the environment and the community (Bouysse‐Cassagne & Harris, 1987). The Aymara worldview pursues a balance with spiritual and material forces, a vision of a renewed nature that inspires contemporary society to locate itself in the cosmos from an ethical position. The Aymara worldview is based on respect, affection and gratitude for the land, water, and all living beings that share habitats (Báez‐Romero, 2017). The concept of diversity acquires a fundamental role in these cultures, as it is associated with socioeconomic and cultural diversity. For the high Andean communities, rituals transmit, reinforce, and deepen ancestral knowledge and practices that warn that the world is alive and inhabited by positive and negative forces (Castro, 2021). These rites are affective exercises, where human desire is directed towards others, such as the mountains themselves (Gómez et al., 1998).

### 5.2 Habits

The Huasco Lake Aymara Indigenous Association (Asociación Indígena Aymara Laguna del Huasco, AIALH, 2005) maintains Aymara pastoral practices as a grazing technique based on the seasonal transhumance of cattle, meaning that it uses different ecological units in accordance with the concentration‐abundance and dispersion‐scarcity of fodder throughout the year. High Andean transhumance patterns are attuned to local variations caused by the combination of ecological units that are particular to each basin (Van Kessel, 1992). Aymara transhumance patterns are organized according to biocultural calendars that are adjusted to climatic variations from year to year (Figure 13). Their flexibility has enhanced practices of domestication and grazing of camelid herds that are compatible with the conservation of high Andean wetlands and a careful management of water. In winter people practice the “floreo,” the ritual involving the community to collectively mark the camelids (Figure 12). Other practices are related to the *Machaq mara* celebration of the new year at the winter's solstice, which means the return of the sun.

### 5.3 Co‐Inhabitants

Central co‐inhabitants in the high Andes are the domesticated camelids alpaca (*Lama pacos*) and llama (*L. glama*). Indigenous Aymara communities have sustained models of life coupled with marked seasonal contrasts, which alternate rain and drought, heat and cold (Marsh, 2015). For this coupling they have resorted to developing biocultural calendars that allow them to adapt to these cycles by closely observing the moon, the sun, and other stars. The sun is observed to interpret annual periods, while the moon is closely monitored to interpret shorter cycles (Lumbreras, 2005). These astronomical calendars are completed by ecological calendars. In the high Andean wetlands, birds are also conspicuous co‐inhabitants, and the arrival and departure of migratory birds such as flamingos mark the beginning of the spring and end of summer. Two species, the Andean flamingo (*Phoenicoparrus andinus*) and the Chilean flamingo (*Phoenicopterus chilensis*), have the world's largest populations in this high Andean region.

### 5.4 Conflicts

Today, increasing uncertainty is linked to climate instability. In the past there were strong interannual oscillations in rainfall, but at a different scale than they are currently (Squeo et al., 2006). However, they were multiannual patterns causing strong contrasts in productivity between very dry years and others that were very rainy, with a lower degree of predictability (Jara et al., 2022). The needs for calendars were essential, especially with agriculture, and the shamans played a fundamental role. Their accumulated knowledge and practices were intertwined with techniques and rituals to face unforeseen climatic changes. Biocultural calendars that covered long‐term periods, beyond 1 year, acquired special relevance. These calendars were based on the knowledge shamans had about the differentiated movements between the sun and the stars. In its daily cycle, the sun moves from east to west. In its annual cycle, from winter to summer, the sun moves from north to south. Following solar time, there are stars that rise earlier and generate new constellations that are associated with changes in weather (Lumbreras, 2005).

Pastoral management carried out in the Salar del Huasco has allowed the maintenance of an animal carrying capacity that has avoided overgrazing (Faúndez, 2004). In contrast, in the Lirima pampas overgrazing is evident (Faúndez, 2009). This degradation could be due to a loss of adaptability of biocultural calendars or to other causes (such as climate change or the recent introduction of cattle, goats and sheep) that have increased since the 1960s. Regarding the first alternative, there have been losses of biocultural calendars associated with losses of the Aymara language by local populations. These losses have altered the synchronization of Aymara cultural practices and nature (Faúndez, 2009). Regarding the second alternative, the introduction of exotic domestic livestock has catalyzed processes of degradation and desertification. In recent decades, this degradation has been exacerbated by the negative impacts of decreased rainfall associated with climate change (González et al., 1991; Maureira, 2021) and unregulated tourism and mining activities (Rocha & Saez, 2003).

Today, the exploitation and pollution of water by mining companies generates severe conflicts with Aymara communities. Northern Chile is rich in minerals and has historically attracted large public and private mining companies that have exerted a growing demand for land, water, and energy to carry out their industrial processes (Romero Toledo et al., 2017). During the military regime in Chile (1973–1990), the 1981 Water Code was signed into law allowing the privatization of water, thus annulling historical customary or usufruct rights to its use (Prieto & Bauer, 2012). Supported by this legislation, mining has impacted the availability of freshwater for Indigenous communities in northern Chile for at least four major reasons.

1. Purchase of surface water rights (Budds, 2009);
2. Exploration and extraction of groundwater without the consent of the communities (Larraín & Poo, 2010; Salinas, 2012; Van Kessel, 1985);
3. Captation of surface water through tubes and wells (Yáñez & Molina, 2011);
4. Contamination of ground and surface water due to high contents of metals or heavy metalloids, such as copper, molybdenum, arsenic, cadmium, mercury, and lead, particularly when mining activities are developed at the head of the basins in areas of abrupt topography and just few kilometers from the communities (Yáñez & Molina, 2008).

Protecting and/or restoring the high Andean wetlands is urgent. Hence, respecting Aymara worldviews and their practices of co‐inhabitation with other‐than‐human species could help to protect Aymara habitats and customary life habits. This biocultural restoration could reverse displacement of Indigenous communities that had to migrate to cities due to the degradation of their habitats (FSP, 2019; Gómez et al., 1998). The promulgation of the Indigenous Law in 1993 incorporated specific rules for the territory and water of Indigenous populations, and should be better enforced. Today, according to the 1993 Indigenous Law, water (privatized in the 1981 Water Code) should be regulated under this legal regime because it recognizes community rights and uses of water (Romero Toledo et al., 2017).

The protection of habitats and access to them by Aymara communities were further strengthened in 1995 with the creation of Indigenous Development Areas (IDA). IDAs are understood as territorial units where the Chilean state supports the protection of common resources and access to them by Indigenous communities with three central goals: to overcome poverty, to preserve Indigenous cultures, and to comply with their demands for land and water. The criteria for the designation of IDAs are based on the ancestral occupation of the territory, high density of Indigenous populations, ecological homogeneity, and dependence on natural resources such as land, water, flora, and fauna. IDAs have helped, but often for the defense of these habitats and water resources, Aymara communities have had to appeal to international conventions, such as the Convention 169 of the International Labour Organization (ILO) and/or the Ramsar Convention (Bolados‐García, 2014a, 2014b).

The protection of habitats has enabled a revitalization of Aymara life habits and ways of thinking. In the Aymara worldview, water is a person who talks and with whom one talks to regenerate life on the farm (Rengifo, 1995). Water is considered as the blood of the Pachamama, a member of the community that is part of rituals along with the spirits of llamas and alpacas (Apaza‐Ticona et al., 2021). Rituals and important dates in the Aymara calendar, such as planting and harvesting, are marked by musical compositions, so that music marks the beat of Aymara time (Duperré, 2021). Music animates ritual events and the memorization of the Aymara worldview. Aware of this shared heritage through oral tradition, today Aymara communities claim the values given to the land and the permanence of their beliefs (Duperré, 2021).

The Aymara worldview and practices bring interpretations of the natural world that might help global society recover the ability to learn from the elements of nature and revitalize awareness of the stars, mountains, water, animals, and plants. This relearning could facilitate the reconnection and synchronization of social and economic practices with natural processes not only in the Aymara territories but also in other mountainous regions of the world.

#### 5.4.1 Links Among Co‐Inhabitants, Their Life Habits and Shared Habitats: A Concise Synthesis

The four biocultural calendars examined in this article exhibit unique environmental characteristics in which different humans and other species attune their life habits to seasonal cycles. Cultural practices and socio‐environmental changes in southwestern South America combine cycles of harvesting, fishing, agriculture, and grazing that are organized in biocultural calendars. These calendars are signaled by the relationships of co‐inhabitation among keystone biocultural species that have coevolved their life habits with human and other‐than‐human co‐inhabitants in the habitats they share (Table 1).

**Table 1:** Synthesis of Interconnected Relationships Among Biocultural Keystone Co‐Inhabitants, Their Life Habits, and Shared Habitats Examined in Four Biocultural Calendars in Chile

Local community/Territory	Habitats	Habits	Co‐inhabitants
Yahgan Indigenous community/Cape Horn Biosphere Reserve	Marine coastal ecosystems	Collection of Chilean king crab and other species of shellfish	Chilean king crab (*Lithodes santolla* (Molina, 1782)), mussels (*Mytilus chilensis*)
	Wetlands	Rushes gathering and basketry	Rushes (*Marsippospermum grandiflorum*), snipe (*Gallinago paraguaiae*).
	Forests	Collection of fungi (*Cyttaria*) and bark of Evergreen beech for the manufacture of handcraft canoes	Katran (*Cyttaria spp*.), Evergreen beech (*Nothofagus betuloides*), agellanic woodpecker (*Campephilus magellanicum* (P.P. King, 1827))
Local fishers/Chiloe archipelagoes	Marine coastal ecosystems	Seaweed harvesting and fishing	Red seaweeds (*Agarophyton chilense (Gracilaria chilensis* C.J.Bird, McLachlan & E.C. Oliveira, 1986))*, Sarcothalia crispata*), Giant kelp (*Macrocystis pyrifera* (Linnaeus) C.Agardh), Chilean sea urchin (*Loxechinus albus*), Patagonian blennie (*Eleginops maclovinus*), Pink cusk‐eel (*Genypterus blacodes* (Forster, 1801))
	Pasture lands	Potato farming and sheep shearing	Potatoes (*Solanum tuberosum* L., 1753), Sheep (*Ovis aries* (Linnaeus, 1758))
Lafkenche‐Williche Indigenous communities/Coastal areas of central southern Chile	Marine coastal ecosystems	Shellfish and seaweed harvesting	Giant kelp (*M. pyrifera*), “cochayuyo” or southern bull kelp (*Durvillaea antarctica*), limpet (*Fissurella spp* Bruguière, 1789).
	Pasture lands	Breeding of cattle, goats and sheep	Sheep (*O. aries),* goat (*Capra hircus* (Linnaeus, 1758)), Cattle (*Bos taurus* (Linnaeus, 1758))
	Family homes	Wool weaving using looms built with native Laurel	Sheep (*O. aries*), *trihue* or “Chilean laurel” (*Laurelia sempervirens* (Ruiz & Pav.) Tul.)
Aymara Indigenous communities/High‐Andes of northern Chile	High‐Andean wetlands (“bofedales”)	Breeding of native camelids and other livestock species of European origin	Llama (*Lama glama*), alpaca (*Vicugna pacos*), sheep (*O. aries*), cows (*B. taurus*), Guayata (*Chloephaga melanoptera* (Eyton, 1838)), Andean flamingo (*Phoenicoparrus andinus*), Chilean flamingo (*Phoenicopterus chilensis*).

## 6 Concluding Remarks

We developed the concept of biocultural calendars using the 3Hs model of the biocultural ethic. This biocultural approach can help us gain a more precise understanding to examine the impacts of climate change on human communities and their ecosystems. To design biocultural conservation programs that contribute to possible forms of mitigation and/or adaptation to climate change, this biocultural approach has a heuristic value for at least five reasons.

First, the concept of *habitat* with its biophysical and symbolic‐linguistic dimensions enhances our understanding about temporal and spatial dimensions of biocultural calendars. Hence, biocultural calendars produce a systemic integration of space and time by combining concepts of the 3Hs model and ecological calendars. This integration is especially relevant for patterns of transhumance of communities and fauna that move along latitudinal or altitudinal gradients, such as the pastoral life habits of Aymara people and their camelids in high Andean habitats (Table 1). This coupling of space and time may be particularly helpful for designing and adapting local practices to climate change trends and variability along altitudinal and latitudinal gradients in other regions of the world.

In the ethnoecological literature, *calendars of the human body* have been reported as expressing a deep understanding of the complex connectivity that the human body has with agricultural activities and ecological processes in mountainous geographies (Kassam et al., 2018). Just as Aymara communities in the Andes of northern Chile migrate with their herds of camelids following temporal and spatial patterns along altitudinal gradients, in the Pamir Mountains of Central Asia agropastoral communities organize their use of pastures according to temporal and spatial patterns of water availability provided by the glacier melt, snow cover, and precipitation (Bulbulshoev et al., 2011; Scheimreif, 2018). These cases of seasonal calendars illustrate how temporal dimensions are integrated with spatial dimensions in the relationships that cultures have with their own human bodies as well as with their habitats. To confront climate change, this spatial‐temporal concept of the habitat has two important implications. (a) As Kassam et al. (2018) have noted it is necessary to reconceptualize a fixed notion of time (e.g., the Gregorian calendar depending solely on fixed astronomical cycles) toward a flexible and relational notion of time that is based on close observation of the habitat and its seasonal indicators, including abiotic (e.g., the first snow or last frost) and biotic (e.g., arrival of migratory birds or the flowering of a plant) phenomena. These indicators respond to climate along spatial (e.g., altitudinal gradients) and temporal (e.g., seasons of the year) gradients enabling dynamic synchronizations of subsistence activities and other life habits. (b) The conservation of habitats and access to them by local communities across spatial and temporal gradients should be a priority in environmental policies in order to maintain these forms of dynamic culture‐nature synchronization, thereby enhancing the capacity to adapt to climate change.

Second, the concept of life *habits* enhances our understanding about how the life cycles of co‐inhabitants are coupled with the annual seasonality of their shared habitats. Life habits are understood as a modus vivendi that gives agency not only to humans but also to other‐than‐human species (Rozzi, 2018, p. 27). For example, gathering rushes and weaving baskets by Indigenous Yahgan handcrafters is attuned to the life cycle of the rushes that are sensitive to changes in temperature and rainfall (Table 1). In turn, the time for gathering rushes is indicated by the arrival of migratory bird species, particularly snipes, as explained in traditional Yahgan stories (Rozzi, Massardo, Anderson, et al., 2010). In this way, the life habits of humans, plants, and birds can be synchronized, even under conditions of climate change.

In the ethnoecological literature, attention has been given to customary laws, customs, and knowledge inherited from elders (humans and other‐than‐humans) (Woodward & Marrfurra‐McTaggart, 2019). This approach converges with our concept of life habits, and how these are embedded in interrelationships with other co‐inhabitants in shared habitats. Just as in the archipelagos of Cape Horn the Yahgan artisans pay attention to the reproductive behavior of the snipes to know when to go to collect reeds, in the wetlands of the Daly River in northwest Australia the life habits of indigenous Ngan'gi communities pay attention to animal behavior, seasonality of water flows in rivers, and plant phenology to determine hunting, fishing, and fruit‐gathering periods (Woodward & Marrfurra‐McTaggart, 2019). This type of habits and seasonal calendars leads to "healthy land, healthy people" results. As in Cape Horn (Box 1), in Australia educational programs have been established for the intergenerational transmission of language, worldview, and seasonal calendars in order to maintain the well‐being of the human community and the group of beings with whom they share the habitats through the inter‐species synchrony of life habits.

Third, the concept of *co‐inhabitants* enhances the identification of biocultural keystone species. Understanding the critical role that biocultural keystone species play for the well‐being of local communities and the conservation of biocultural diversity is essential for the design of policies or practices for adaptation to or mitigation of climate change, and more broadly of social‐environmental change. For example, the life cycle of the seaweed *Sarcothalia crispata* has become the focus of novel management programs designed by fishers and scientists in the archipelagoes of Chiloe (Avila et al., 1996). In the 1980s, this seaweed was initially exploited as a mere raw material for the production of carrageenan. Working with women from fisher communities, this *S. crispate* was identified as a biologically, economically, and culturally important species, and sustainable seaweed‐harvesting programs were subsequently established with a precise but flexible calendar for repopulation, cultivation, harvesting, and commercial activities (IFOP, 2013). *Biocultural keystone species* also can be recognized as flagship species for conservation. Confronted with the complexities of climate change, the focus on certain species appeals to and facilitates the design of management and biocultural conservation programs (Arango et al., 2007; Dietz et al., 1994). Importantly, calling these species “co‐inhabitants” implies an ethical consideration for their intrinsic value and instills a sense of responsibility for the well‐being of human and other‐than‐human co‐inhabitants, their habits, and shared habitats (Rozzi, 2015b).

In the ethnoecological literature, the concept of *cultural keystone species* (in Nabhan & Carr, 1994) has been used to identify species of exceptional significance to a socio‐cultural group due to their prevalence in language, ceremonies, diet, medicines, symbolic presence in traditional stories, and/or their use as seasonal or phenological indicators (Cristancho & Vining, 2004; Garibaldi & Turner, 2004). An illustrative example of a phenological indicator species is found among the Tsimshian Indigenous people in the Pacific Northwest Coast. Just as in the Chiloe archipelagos the seaweed *Sarcothalia crispata* is key to the local economy, in the Pacific Northwest the seaweed “Red laver” (*Pyropia abbottiae* (V.Krishnamurthy) S.C. Lindstrom, 2011) is the most eaten red algae (Turner, 2003). The exact harvesting time of this seaweed is indicated by phenological cues of the stinging nettles (*Urtica dioica* L.). Tsimshian people watch the stalks of the stinging nettle mature and elongate; as they grow in the coastal habitats, so do the seaweed fronds grow in the intertidal zone. The red laver is harvested in late spring, generally in May, so much that in the Tsimshian language this month is called ‘‘the month of the seaweed’’ (Turner, 2016). Today, as in the past, the seaweed is an important component of Tsimishians' diets and traditional life ways, because harvesting and processing bring families and communities together, thus providing opportunities for learning and teaching stories, songs, language, and knowledge about weather patterns, tides and currents as well as of the growth and usable life stages of the seaweed; and optimum drying locations and techniques to achieve the best flavors and greatest nutritional value (Turner & Turner, 2008). This example illustrates how biological and cultural dimensions are interwoven in the relationships that Tsmishians have with the Red laver, the stinging nettles and their habitats. For this reason, in the past (Ibarra et al., 2012; Rozzi, Massardo, Anderson, et al., 2010), and in this article, rather than cultural keystone species we have used the term biocultural keystone species.

Fourth, a conceptual understanding of *conflicts* is necessary to maintain or restore biocultural calendars in the context of climate change and complex social‐environmental problems. As documented above, the four indigenous communities considered in our analysis are confronted with conflicts that include multiple ecological, social, and cultural dimensions For example, in the high Andean wetlands, Aymara families aim to carefully manage delicate water balances through a system of nomadic herding of camelids (whose demand for water is low), but including today cattle of European origin (with higher water requirements). These nomadic habits are intertwined in a complex scenario with other uses of water, principally mining. Mining activities have degraded wetlands and their biodiversity, and triggered accelerated depopulation of Andean rural areas along with disruption of biocultural calendars.

To address this type of conflicts, a *biocultural conservation* approach contributes to balancing the interests and needs among different stakeholders. Just as the Chilean state has created Indigenous Development Areas (IDAs) to protect biological and cultural diversity, worldwide international programs have embraced the concept of including human communities and their habitats in conservation programs (e.g., Man and Biosphere Program, Guevara & Laborde, 2008) and have highlighted the interrelated values of natural and cultural heritages (e.g., UNESCO World Cultural and Natural Heritage Convention; UNESCO, 1992; Bridgewater & Rotherham, 2019). These protected areas and associated programs provide alternatives to polarized positions and offer opportunities for conflict resolution (Castro & Romo, 2006; Romero‐Toledo, 2001). Biocultural conservation approaches could be strengthened by using biocultural calendars to facilitate dialogues and exchanges of knowledge among stakeholders with contrasting socioeconomic interests that concur in current complex communities made up of members from indigenous and other local communities as well as of members of urban societies who are all affected by climate change.

Fifth, the four former concluding remarks show how the concept of *biocultural calendars* integrates concepts of ecological calendars and the biocultural ethic to cope with social and environmental dimensions of climate change. The drivers of climate change are of two types: direct and indirect (MEA, 2005). Direct drivers (or proximate causes) mainly include technological processes and biophysical changes, while indirect drivers (or ultimate causes) involve cultural and social dimensions. Direct drivers have been much more studied than indirect ones. The latter carry an essential ethical dimension to address the ultimate causes of climate change, and more broadly current socio‐environmental problems (Herman, 2016). In this article we offer the conceptual framework of the biocultural ethic that has been developed in Chile but could have broader applications for addressing indirect drivers of climate change. The “3Hs” model prioritizes the connections between biological and cultural diversity to guide the habits of global society in a way that could avoid further biocultural homogenization causing suffering or extermination of myriads of co‐inhabitants and could instead foster biocultural conservation.

## Conflict of Interest

The authors declare no conflicts of interest relevant to this study.

## Data Availability

This is a review paper; no data were used in it.
